# Structural Characterization, Antimicrobial Activity and BSA/DNA Binding Affinity of New Silver(I) Complexes with Thianthrene and 1,8-Naphthyridine

**DOI:** 10.3390/molecules26071871

**Published:** 2021-03-26

**Authors:** Darko P. Ašanin, Sanja Skaro Bogojevic, Franc Perdih, Tina P. Andrejević, Dusan Milivojevic, Ivana Aleksic, Jasmina Nikodinovic-Runic, Biljana Đ. Glišić, Iztok Turel, Miloš I. Djuran

**Affiliations:** 1 Department of Science, Institute for Information Technologies Kragujevac, University of Kragujevac, Jovana Cvijića bb, 34000 Kragujevac, Serbia; darko.asanin@uni.kg.ac.rs; 2 Institute of Molecular Genetics and Genetic Engineering, University of Belgrade, Vojvode Stepe 444a, 11042 Belgrade, Serbia; sanja.bogojevic@imgge.bg.ac.rs (S.S.B.); dusan.milivojevic@imgge.bg.ac.rs (D.M.); ivana_aleksic@imgge.bg.ac.rs (I.A.); 3 Department of Chemistry and Biochemistry, Faculty of Chemistry and Chemical Technology, University of Ljubljana, Večna pot 113, SI-1000 Ljubljana, Slovenia; Franc.Perdih@fkkt.uni-lj.si; 4 Department of Chemistry, Faculty of Science, University of Kragujevac, R. Domanovića 12, 34000 Kragujevac, Serbia; tina.andrejevic@pmf.kg.ac.rs; 5 Serbian Academy of Sciences and Arts, Knez Mihailova 35, 11000 Belgrade, Serbia

**Keywords:** silver(I) complexes, thianthrene, 1,8-naphthyridine, antimicrobial activity, *Caenorhabditis elegans*, DNA/BSA interaction

## Abstract

Three new silver(I) complexes [Ag(NO_3_)(tia)(H_2_O)]*_n_* (**Ag1**), [Ag(CF_3_SO_3_)(1,8-naph)]*_n_* (**Ag2**) and [Ag_2_(1,8-naph)_2_(H_2_O)_1.2_](PF_6_)_2_ (**Ag3**), where tia is thianthrene and 1,8-naph is 1,8-naphthyridine, were synthesized and structurally characterized by different spectroscopic and electrochemical methods and their crystal structures were determined by single-crystal X-ray diffraction analysis. Their antimicrobial potential was evaluated against four bacterial and three *Candida* species, and the obtained results revealed that these complexes showed significant activity toward the Gram-positive *Staphylococcus aureus,* Gram-negative *Pseudomonas aeruginosa* and the investigated *Candida* species with minimal inhibitory concentration (MIC) values in the range 1.56–7.81 μg/mL. On the other hand, tia and 1,8-naph ligands were not active against the investigated strains, suggesting that their complexation with Ag(I) ion results in the formation of antimicrobial compounds. Moreover, low toxicity of the complexes was detected by in vivo model *Caenorhabditis elegans*. The interaction of the complexes with calf thymus DNA (ct-DNA) and bovine serum albumin (BSA) was studied to evaluate their binding affinity towards these biomolecules for possible insights into the mode of antimicrobial activity. The binding affinity of **Ag1**–**3** to BSA was higher than that for DNA, indicating that proteins could be more favorable binding sites for these complexes in comparison to the nucleic acids.

## 1. Introduction

Infections caused by bacteria and fungi threaten human health on a daily basis because many microbes have developed resistance to the commercial antimicrobial agents over time [1]. It is estimated that antimicrobial resistance causes at least 50 thousand deaths each year in Europe and the United States and that it will cause 10 million deaths worldwide per year by 2050 [2]. Considering this, the problems related to the microbial infections and resistance occurrence demand urgent and effective solution, which is based on the development of novel antimicrobial agents. It is well known that silver(I) compounds have shown significant antimicrobial activity, such as silver(I) nitrate which was used for the prevention of eye infections in newborns [3], and silver(I) sulfadiazine (AgSD) applied for the treatment of bacterial infections in burns [4]. Silver(I) complexes have also shown antiproliferative [5] and antiviral [6,7] activities, exhibiting acceptable toxicity towards human cells [8,9]. The mechanism of antimicrobial activity of the silver(I) complexes is explained by their slow release of Ag(I) ions [10,11]. These ions can interact with the bacterial cell surfaces, enabling their penetration into the cell. Once inside the cell, Ag(I) ions could interact with the biomolecules in its interiors, such as DNA and proteins. They can also cause the production of reactive oxygen species (ROS), leading to bacterial cell death [10,11]. Due to this multi-targeting mechanism, the silver(I) complexes act as antimicrobial agents, which might overcome the problem of bacterial resistance [12].

Depending on the type of heteroatom present in heterocyclic compounds, they may show a wide spectrum of pharmacological activities [13,14,15,16,17]. For instance, the United States Food and Drug Administration (FDA) approved several heterocycles containing sulfur or/and nitrogen donor atoms, such as raloxifene as anticancer [18], rosiglitazone as anti-diabetes [19], thiabendazole and clotrimazole as antifungal [20,21], and chloroquine as antimalarial agent [22]. Considering this, these compounds represent the important class of ligands for the synthesis of biologically active silver(I) complexes. For example, the silver(I) complexes with aromatic nitrogen-containing heterocycles (*N*-heterocycles), such as pyridazine, pyrimidine, pyrazine, phthalazine, quinazoline, quinoxaline, and phenazine, have shown significant activity against the Gram-negative bacterium *Pseudomonas aeruginosa* [23,24,25]. On the other hand, an excellent ability to inhibit the growth of *Candida* strains has been observed for the silver(I) complexes with phenanthrolines [26,27,28,29,30] and 1,5-naphtyridine [31]. The significant antimicrobial activity has also been manifested by silver(I) complexes with *N*-methylbenzothiazole-2-thione (mbtt), [(mbtt)_2_Ag(*μ*-mbtt)_2_Ag(mbtt)_2_](NO_3_)_2_ and [Ag(mbtt)_3_]CF_3_SO_3_ against certain Gram-negative (*Xanthomonas campestris*, *Escherichia coli*) and Gram-positive (*Bacillus subtilis*, *Bacillus cereus*, *Staphylococcus aureus*) bacteria [32]. Similarly, silver(I) complexes with *N*-substituted imidazolidine-2-thiones, purine-6-thione, 2-thiouracil, pyrimidine-2-thione, and pyridine-2-thione have shown moderate to good antimicrobial activity against Gram-positive (methicillin-resistant *Staphylococcus aureus* MRSA and *Staphylococcus aureus*), and Gram-negative (*Staphylococcus epidermidis*, *Enterococcus faecalis*, *Shigella flexneri*) bacteria and a yeast *Candida albicans* [33]. 

Considering all these facts, in the present study, we used two heterocycles, thianthrene (tia; *S*-heterocycle) and 1,8-naphthyridine (1,8-naph; *N*-heterocycle) for the synthesis of three new silver(I) complexes [Ag(NO_3_)(tia)(H_2_O)]*_n_* (**Ag1**), [Ag(CF_3_SO_3_)(1,8-naph)]*_n_* (**Ag2**) and [Ag_2_(1,8-naph)_2_(H_2_O)_1.2_](PF_6_)_2_ (**Ag3**). The synthesized complexes were characterized by spectroscopy (NMR, IR, and UV-Vis), mass spectrometry, cyclic voltammetry, and single-crystal X-ray diffraction analysis. These complexes were further evaluated for their in vitro antimicrobial activity and in vivo toxicity in *Caenorhabditis elegans*, an important animal model for rapid toxicity assessment of the novel compounds [34]. The interactions of the complexes **Ag1**–**3** with calf thymus DNA (ct-DNA) and bovine serum albumin (BSA) were studied with the aim to check their binding affinity towards these potential biological targets.

## 2. Results

### 2.1. Synthesis and Characterization of Silver(I) Complexes **Ag1**–**3**

**Scheme 1:**
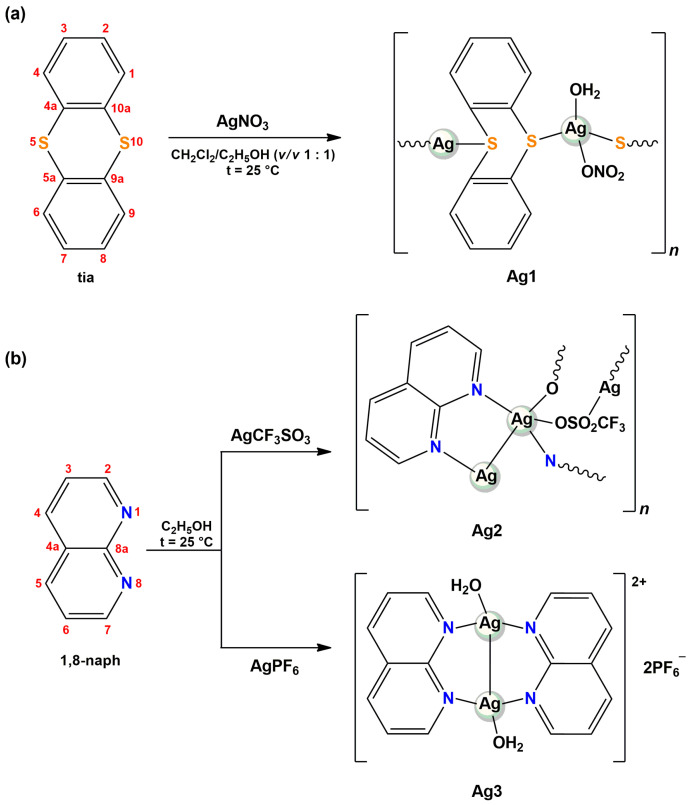
Schematic presentation of the reaction route for the synthesis of silver(I) complexes with tia (thianthrene) (**a**) and 1,8-naph (1,8-naphthyridine) (**b**). Numeration of atoms in the ligands is in accordance with IUPAC recommendations for fused ring systems and used for NMR characterization of complexes **Ag1**–**3**.

Silver(I) complexes **Ag1**–**3** were synthesized according to the route presented in Scheme 1. The reaction of AgNO_3_ with an equimolar amount of thianthrene (tia) in ethanol/dichloromethane (*v/v* 1:1) at ambient temperature yielded polynuclear [Ag(NO_3_)(tia)(H_2_O)]*_n_* complex (**Ag1**), while by reacting 1,8-naphthyridine (1,8-naph) with AgCF_3_SO_3_ and AgPF_6_ in ethanol, polynuclear [Ag(CF_3_SO_3_)(1,8-naph)]*_n_* (**Ag2**) and dinuclear [Ag_2_(1,8-naph)_2_(H_2_O)_1.2_](PF_6_)_2_ (**Ag3**) complexes, respectively, were formed. NMR (^1^H and ^13^C), ultraviolet–visible (UV-Vis) and IR spectroscopy, mass spectrometry, and cyclic voltammetry were applied for characterization of the obtained complexes, while a single-crystal X-ray diffraction analysis was used for the determination of their structures.

#### 2.1.1. Solid-State Characterization

**Figure 1:**
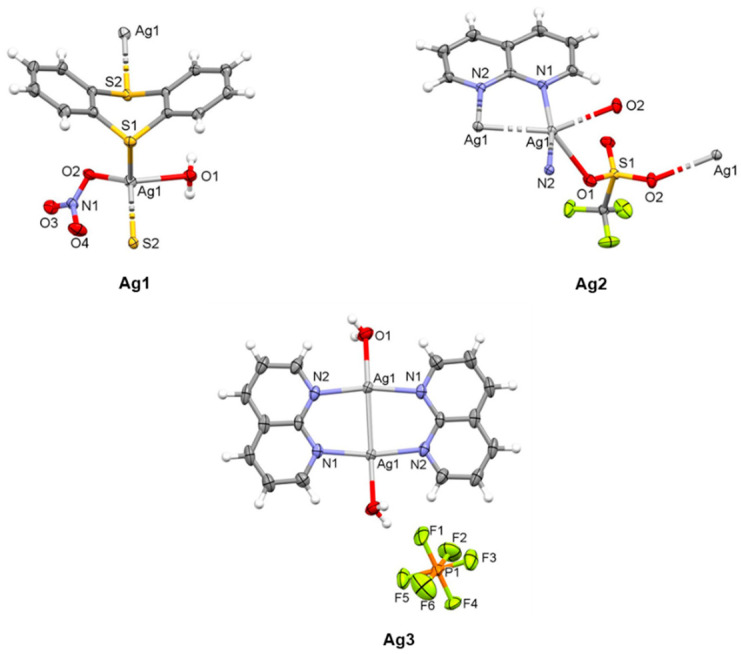
Crystal structures of complexes **Ag1**–**3**. Thermal ellipsoids are drawn at the 50% probability level. For **Ag3**, only one anion is presented for clarity.

**Figure 2:**
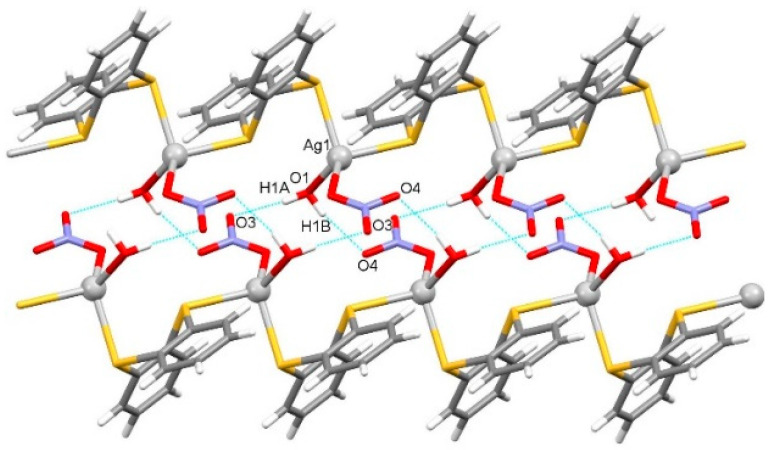
Hydrogen bonded belt in **Ag1**. Hydrogen bonds are drawn by dashed blue lines.

Silver(I) complexes **Ag1** and **Ag2** are polynuclear species with infinite chains and complex **Ag3** is dinuclear species (Figure 1). Complex **Ag1** has a polynuclear structure with tetrahedrally coordinated Ag(I) ion via two thianthrene sulfur atoms (Ag–S distances 2.6198(7) and 2.6747(8) Å), one nitrate oxygen atom (Ag–O distance 2.328(2) Å) and one water oxygen atom (Ag–O distance 2.360(2) Å) (Figure 2, Table 1). Thianthrene acts as a bridging ligand. Hydrogen bonding between coordinated water molecules of one polynuclear chain with the nitrato ligand of the adjacent polynuclear chain enables the formation of an infinite belt. The polynuclear belt is supported also by C3–H3···O1 interactions (Table 2). Belts are connected into a supramolecular structure through C5–H5···O2/O4 interactions.

**Figure 3:**
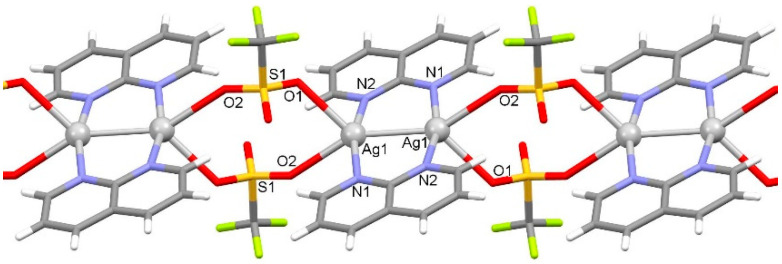
Polynuclear chain in **Ag2**.

Complex **Ag2** has a polynuclear structure with Ag_2_(1,8-naph)_2_ units connected into a chain via bridging triflato ligands (Figure 3). Within the Ag_2_(1,8-naph)_2_ unit, the 1,8-naph is a bridging ligand with Ag–N distances of 2.200(2) and 2.218(2) Å, and this unit is supported also by argentophilic interaction with Ag1···Ag1^i^ distance of 2.7871(5) Å representing the 81% of the sum of van der Waals radii (Table 1). Two triflato ligands connect two adjacent Ag_2_(1,8-naph)_2_ units into a chain through an Ag1–O1 bond distance of 2.555(2) Å and through a somewhat longer Ag1–O2 contact of 2.645(2) Å. The polynuclear chain is supported also by C1–H1···O2 and C7–H7···F2 interactions (Table 2). The crystal structure is stabilized by π···π interactions between 1,8-naph rings of adjacent chains with a centroid-to-centroid distance of 3.6330(17) Å and ring slippage of 1.061 Å as well as by C8–H8···O3 interactions.

Complex **Ag3** has a dinuclear structure with [Ag_2_(1,8-naph)_2_(H_2_O)_1.2_]^2+^ cation and two PF_6_^−^ counter-anions. In [Ag_2_(1,8-naph)_2_(H_2_O)_1.2_]^2+^ cation, the 1,8-naph acts as a bridging ligand with almost identical Ag–N distances (2.186(3) and 2.184(3) Å) and, also, this unit is supported by argentophilic interaction with Ag1···Ag1^i^ distance of 2.7235(6) Å representing the 79% of the sum of van der Waals radii (Table 1). The Ag(I) metal center is coordinated also by a water molecule with Ag–O distance of 2.449(4) Å. However, the water molecule was found to possess a 0.60 occupancy ratio. The crystal structure is stabilized by π···π interactions between 1,8-naph rings of adjacent cations with a centroid-to-centroid distance of 3.602(3) Å and ring slippage of 1.035 Å as well as by various O–H···F and C–H···F interactions (Table 2).

The IR spectroscopic data of the complexes **Ag1**–**3** are following their structures determined by a single-crystal X-ray diffraction analysis. The IR spectra of complexes **Ag1** and **Ag3** exhibited a broad band at ~ 3440 cm^−1^ due to the stretching vibration of the –OH group, confirming the presence of coordinated water to Ag(I) ion in these complexes. The nitrate coordination in the **Ag1** complex is confirmed by a very strong band at 1384 cm^−1^ with a tendency to split into two bands [35], while in the case of **Ag2**, strong bands in the 1300–1000 cm^−1^ region originate from a coordinated triflate in this complex [36,37]. More precisely, the bands at 1163 and 1134 cm^−1^ can be attributed to the symmetric and asymmetric stretching vibrations of the –CF_3_ group, while the bands at 1254, and 1044 and 1032 cm^−1^ are due to the asymmetric and symmetric stretching of the –SO_3_ group in triflate, respectively [36]. Regarding the **Ag3** complex, the presence of a strong band at 831 cm^−1^ assigned to the stretching vibrations of the PF_6_^−^ is a consequence of its existence as a counter-anion in the crystal lattice of this complex [38,39]. 

#### 2.1.2. Solution Behavior

The NMR spectra of the synthesized silver(I) complexes and corresponding ligands (Supplementary Materials) were assigned based on the previously reported NMR spectroscopic data for tia [40] and 1,8-naph [41]. ^1^H- and ^13^C-NMR spectra of the presently investigated silver(I) complexes contain the same number of signals as those for the uncoordinated tia (**Ag1**) and 1,8-naph (**Ag2** and **Ag3**) ligands, being in accordance with the bidentate bridging coordination mode of these ligands to Ag(I) ion. While in the case of **Ag1** complex, the resonances in both ^1^H- and ^13^C-NMR spectra are almost unshifted in respect to those for the uncoordinated tia ligand (vide infra in Materials and Methods section), the chemical shifts of ^1^H- and ^13^C-NMR resonances for **Ag2** and **Ag3** differ significantly from those of 1,8-naph. All ^1^H resonances for coordinated 1,8-naph in **Ag2** and **Ag3** are downfield shifted with respect to those for the free ligand. The chemical shifts of ^1^H-NMR resonances in these complexes are strongly dependent on the proton position with respect to the nitrogen donor atoms. As can be expected, the largest coordination shift (Δ*δ* = *δ*_complex_ – *δ*_1,8-naph_) of + 0.32 (for both complexes) were observed for H2/H7 protons, which are adjacent to the nitrogen atoms. On the other hand, there is no strict rule in the shifting of ^13^C resonances of the silver(I) complexes **Ag2** and **Ag3** with respect to the carbon position relative to the nitrogen atoms. 

In the mass spectra of the investigated silver(I) complexes, the major doublet peaks are those centered at *m*/*z* = 540.9176 (**Ag1**) and 367.0107 (**Ag2** and **Ag3**), which are consistent with the presence of mononuclear [Ag(tia)_2_]^+^ and [Ag(1,8-naph)_2_]^+^ cations, respectively. As it was found previously, the mass spectra of silver(I) complexes show doublet peaks with almost equal intensity for a mononuclear species or triplet peaks with approximately 1:2:1 intensity ratios for dinuclear silver(I) species, as a consequence of the nearly equal abundance of two silver isotopes, ^107^Ag (51.84%) and ^109^Ag (48.16%) [29].

The UV-Vis spectra of the complexes **Ag1**–**3**, recorded in dimethyl sulfoxide (DMSO), are similar to those of the uncoordinated tia and 1,8-naph ligands, suggesting that the absorption maxima (λ_max_) at 259 nm for **Ag1** and 307 nm for **Ag2** and **Ag3** are due to the characteristic π→π* transitions in the corresponding heterocyclic ligand [42,43]. In order to check the stability of complexes **Ag1**–**3**, their UV-Vis spectra were also recorded 24 h and 48 h after dissolution. As an illustration, time-dependent UV-Vis spectra of the **Ag3** complex were shown in Figure S1. For **Ag2** and **Ag3**, the observed transitions remained unmodified over 48 h at room temperature, implying their stability in the solution during this time. Nevertheless, a slight decrease in the intensity of the absorption maximum of 12% was noticed for **Ag1**, although without significant modifications of the spectrum shape and position of the absorption maxima, indicating that this complex is the least stable amongst the synthesized silver(I) complexes. 

With the aim to investigate the air/light stability of complexes **Ag1**–**3**, which is important for their possible external application in the form of ointments, gels, and coating materials of dressings [44], sterile cellulose discs impregnated with their DMSO solutions were exposed to air and light for 48 h. The obtained results were compared with those for silver(I) salts used for the synthesis of the complexes (AgNO_3_, AgCF_3_SO_3,_ and AgPF_6_) (Figure S2). As can be seen, all investigated silver(I) complexes were less darkened than the salts during 48 h, indicating the slightly higher air/light stability of the complexes **Ag1–3**. Among the complexes, **Ag1** turned out to be the least stable under the investigated experimental conditions. 

**Figure 4:**
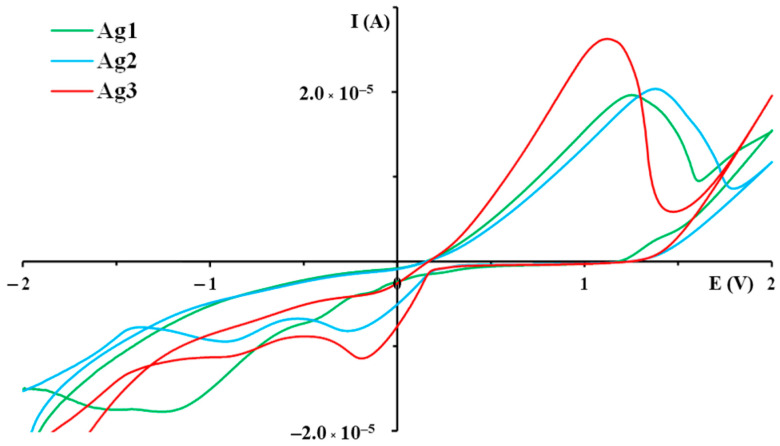
Cyclic voltammograms of the silver(I) complexes **Ag1**–**3** at GC electrode in DMSO and 0.1 M TBAHP as a supporting electrolyte with a scan rate of 50 mV/s. The conditions are given as follows: E_begin_ = −2.0 V, E_end_ = 2.0 V and E_step_ = 0.002 V.

The electrochemical behavior of the metal complexes is highly relevant for a better understanding of their stability and biological activity, as well as the way of interaction with biomolecules [45,46]. In accordance with this, the cyclic voltammograms (CV) of complexes **Ag1**–**3** were recorded at the glassy carbon (GC) electrode in DMSO and 0.1 M tetrabutylammonium hexafluorophosphate (TBAHP) as a supporting electrolyte under the following conditions, E_begin_ = −2.0 V and E_end_ = 2.0 V (Figure 4). As can be seen from this figure, a broad anodic peak, which can be attributed to Ag(I)→Ag(II) oxidation process, was observed [47,48]. When the cyclic voltammogram was recorded in the cathodic direction, two distinctive reduction peaks were detected for all complexes (Table 3), which were assigned to Ag(II)→Ag(I) and Ag(I)→Ag(0) reduction processes, in accordance with the other silver(I) complexes where electrochemical behavior was previously investigated [47,49]. 

### 2.2. Biological Evaluation of the Silver(I) Complexes **Ag1**–**3**

#### 2.2.1. Antimicrobial and Antiproliferative Effect of Silver(I) Complexes **Ag1**–**3**

Given the traditional antimicrobial properties shown by silver(I) complexes [23,24,25,26,27,28,29,30,31,32,33], the activity of silver(I) complexes **Ag1**–**3** and the ligands, tia and 1,8-naph used for their synthesis, was determined against two Gram-positive (*S*. *aureus* and *L. monocytogenes*), two Gram-negative (*E. coli* and *P. aeruginosa* ATCC 10332 and BK25H [50]) bacteria, and three *Candida* species (*C. albicans*, *C. krusei,* and *C. parapsilosis*; Table 4). The antimicrobial activity of different AgX salts against these bacterial and fungal strains was previously evaluated [25]. Although these salts have shown significant antimicrobial activity, their use has been limited due to their precipitation in the form of AgCl under physiological conditions. As a consequence of this process, Ag(I) ions could not reach the infected sites and manifest antimicrobial activity [10]. 

Overall, presently investigated silver(I) complexes **Ag1**–**3** showed significant antimicrobial activity toward the Gram-positive *S. aureus,* two Gram-negative *P. aeruginosa* species, and tested *Candida* species, while the lower sensitivity was detected in the case of *L. monocytogenes* and *E. coli* (Table 4). The most sensitive species was *C. krusei*, with MIC values being 1.56 µg/mL for all tested silver(I) complexes (3.6, 4.0, and 1.0 µM for **Ag1**–**3**, respectively). On the other hand, both tia and 1,8-naph ligands did not affect the microbial growth at 200 µg/mL (925 and 1537 µM, respectively), suggesting that the observed activity of the complexes is due to the presence of Ag(I) ions. As suggested previously, the antimicrobial activity of silver(I) complexes are connected to the complex ability to release free Ag(I) ions that may exert their antimicrobial action through different pathways [11]. On the other hand, the antiproliferative effect of these complexes was considerably higher but comparable to the observed MIC values (Table 4). On some level, this may limit the antimicrobial application of these complexes, but it is worth mentioning that silver(I) complexes with different classes of ligands have been studied for their antitumor potential and some of them showed antiproliferative activities higher than cisplatin [51].

These results are in line with our previous work in which we showed that the silver(I) complexes with a 1,5-naphthyridine ligand, which is a structural isomer of 1,8-naph, have remarkable activity against a range of *Candida* species, while for the tested bacterial species, MIC values obtained in this study are 2–3 times lower than in our previously published work (Table 4) [31]. Considerable anti-*Candida* activity has been also reported for the silver(I) complexes with 1,7- and 4,7-phenanthroline [29,30], while, on the other hand, silver(I) complexes with metronidazole (mtz), [Ag(mtz)_2_(NO_3_)] and [Ag_2_(mtz)_4_](BF_4_)_2_, showed better activity against the tested bacterial species and very low activity against *C. albicans* [44].

The different antibacterial behavior of silver(I) complexes obtained in this study might be related to their mode of action [52]. Gram-positive and Gram-negative bacteria have a different peptidoglycan cell wall and therefore, it can be assumed that the different bactericidal behavior of the silver(I) complexes can be explained by their ability to interact with the cell wall and pass through the membrane causing bacterial growth inhibition [52,53,54]. 

#### 2.2.2. *C. elegans* Toxicity

**Figure 5:**
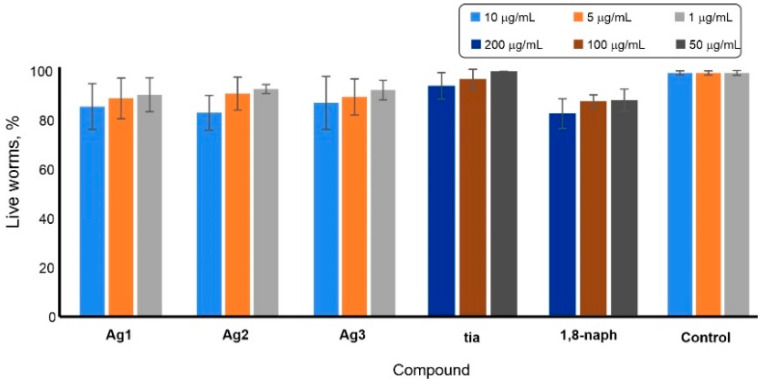
Survival of *C. elegans* in the presence of silver(I) complexes **Ag1**–**3** alongside tia and 1,8-naph ligands (%, average ± the SD) after 48 h of treatment.

A genetically tractable, multicellular nematode *C. elegans*, is a widely used model organism as a high throughput platform for toxicity assessment and more recently for the discovery of new antimicrobial compounds [55,56]. The tested complexes **Ag1**–**3** and the corresponding ligands showed low toxicity against *C. elegans* in concentrations relevant to the observed MICs and cytotoxicity on MRC-5 cells (Figure 5 and Table 4). The highest percentage of dead worms was observed upon treatment with the highest concentrations of **Ag2** complex and 1,8-naph, being 17 and 18%, respectively (Figure 5). The moderate toxicity against *C. elegans* was also previously observed for silver(I) complexes with different pyridine-4,5-dicarboxylate esters as ligands, which showed significant activity on the cow mastitis-associated pathogens [49]. The differences in the two toxicity models are possibly due to the more complex uptake system present in nematode in comparison to the direct application to cells in in vitro model.

### 2.3. BSA Binding Study

Serum albumin (SA) is one of the most abundant proteins present in the blood plasma and plays a crucial function in the transport of various endo- and exogenous compounds, including pharmacologically used agents [57]. Therefore, the distribution, free concentration, and metabolism of these agents can be changed as a result of their binding to serum albumin. Considering this, the study of the binding interaction of this protein with potential therapeutic agents could contribute to an understanding of the mechanism of their transport and distribution in the human body and identification of the dynamics of their action [57]. Bovine serum albumin (BSA) has been widely used as a model protein for this purpose due to its structural similarity with human serum albumin (HSA) of approximately 76%, low cost, and some unusual binding properties [57,58]. Fluorescence spectroscopy is one of the most convenient methods for the investigation of BSA binding interactions [59]. The intrinsic fluorescence of this protein is mainly due to tryptophan (Trp), tyrosine (Tyr), and phenylalanine (Phe) residues, whereas the binding interaction between the investigated compound and BSA will cause fluorescence quenching [59]. 

**Figure 6:**
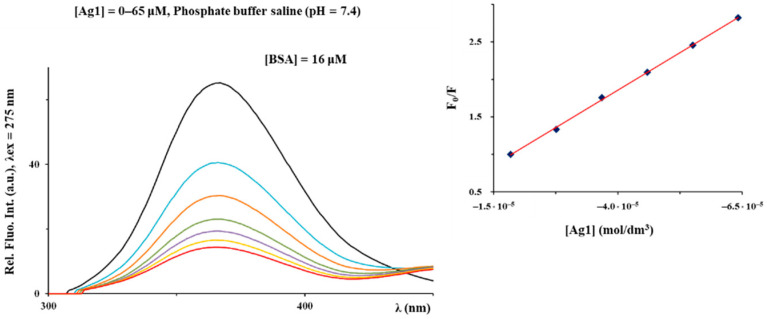
Fluorescence emission spectra of BSA in the presence of an increasing amount of **Ag1** complex. The arrow shows the intensity changes upon the gradual addition of the complex. Inserted graph: Stern-Volmer plots of F_0_/F vs. complex.

The emission spectra of BSA were recorded in the absence and presence of an increasing amount of the investigated silver(I) complexes **Ag1**–**3**. In all cases, a remarkable quenching of a fluorophore was observed with the gradual addition of the complex, indicating that its interaction with BSA occurred [57,58,59,60,61]. As can be seen from Figure 6, upon addition of an increasing concentration of **Ag1** complex to BSA solution of a constant concentration, a significant decrease of fluorescence intensity at 367 nm was observed, leading to the formation of a BSA-complex system. 

In order to study the quenching mechanism, Stern-Volmer and Scatchard equations were applied for the analysis of the fluorescence quenching data and the obtained data (Stern-Volmer constants (*K_sv_*), quenching rate constants (*K_q_*), binding constants (*K_A_*), and the number of binding sites per BSA (*n*)) are presented in Table 5. The values of *K_sv_* constants indicate that the **Ag1** complex containing tia ligand has a higher affinity for BSA compared to the remaining two complexes **Ag2** and **Ag3** with 1,8-naph ligand. The *K_sv_* values for **Ag2** and **Ag3** are similar to those calculated for silver(I) complex with 1,2-bis(4-pyridyl)ethene [62], as well as for those with *N*-methyl-1,3,5-triaza-7-phosphaadamantane and tris(pyrazol-1-yl)methanesulfonate [63]. The *K_q_* constant depends on the probability of a collision between fluorophore and quencher and is a measure of the tryptophan residues exposure to the investigated compound [64]. As can be seen from Table 5, the *K_q_* values for the complexes **Ag1**–**3** are in accordance with their good quenching ability of the BSA fluorescence. The *K_q_* values for all investigated complexes are significantly higher than 2 × 10^10^ M^−1^·s^−1^ (the value of maximum diffusion collision quenching rate constant), indicating the static quenching mechanism [63,65]. The *K_A_* values for **Ag1**–**3** are in an optimal range, i.e., these constants are high enough to suggest that the complexes can be efficiently carried in the blood (which is an important property of a drug [57]), but not so high to prevent their release from the BSA upon arrival to the target cell [66]. Finally, the calculated *n* values for complexes suggest their binding to only one binding site per protein molecule.

### 2.4. Lipophilicity Assay

The lipophilicity of a compound is in accordance with its cellular uptake efficiency and is of great importance for the new drug candidate design [67,68]. The octanol-water partition coefficient (*log*P) is a measure of lipophilicity and can be determined by the flask-shaking method [69]. Thus, the *log*P values for complexes **Ag1**–**3** are 0.72, −0.14, and 0.37, respectively, being in accordance with the *log*P values range of −0.4 to 5.6 for new pharmacophores in the comprehensive medicinal chemistry [70]. The greater *log*P values of complexes **Ag1** and **Ag3** in respect of **Ag2** indicate that these two complexes are mainly distributed in the octanol phase, further implying their higher cellular uptake efficiency.

### 2.5. DNA Interaction

**Figure 7:**
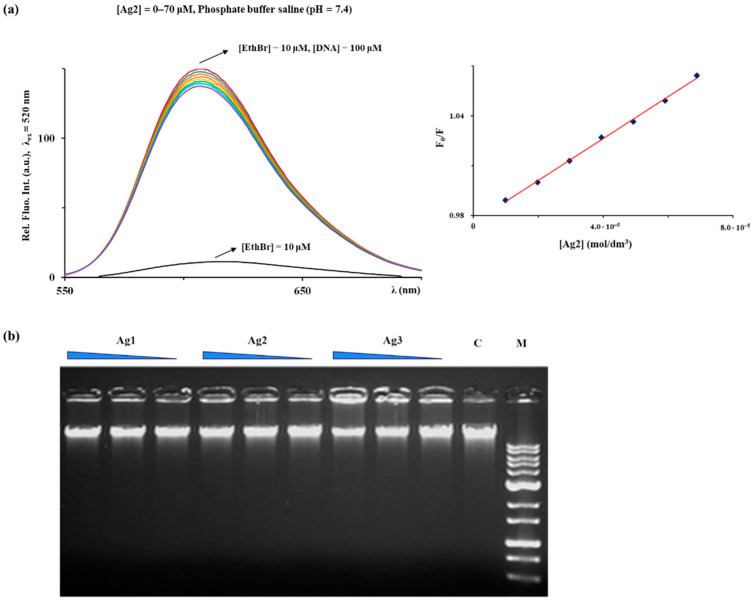
(**a**) Fluorescence emission spectra of EthBr–DNA system in the presence of an increasing amount of **Ag2** complex. The arrow shows the intensity changes upon the gradual addition of the complex. Inserted graph: Stern-Volmer plots of F_0_/F vs. complex. (**b**) Interaction of silver(I) complexes **Ag1**–**3** with commercial lambda bacteriophage DNA assessed by gel electrophoresis. 0.8% agarose gel with EthBr against a HyperLadder™ 1 kb DNA Ladder plus (FastGene) (Mw), DMSO is a control sample (C). Gel visualization by the Gel Doc EZ system (Bio-Rad, Life Sciences, Hercules, USA), equipped with the Image Lab™ Software.

Interactions of metal ions and their complexes with DNA are of great importance for the design of novel metal-based therapeutic agents [71]. Moreover, it is known that the mechanism of antimicrobial activity of silver(I) complexes can be related to their interaction with different cellular biomolecules, including DNA [10]. Firstly, the binding affinity of **Ag1**–**3** complexes toward DNA was investigated by fluorescence spectroscopy by performing competitive binding experiments based on the displacement of ethidium bromide (EthBr) from DNA. It is well known that EthBr intercalates between adjacent base pairs in the DNA double helix resulting in the enhancement of its fluorescence [63]. After the addition of the investigated complex, a decrease in the fluorescence intensity of the EthBr-DNA system will occur if this complex intercalates into DNA or after its binding to EthBr-DNA, which leads to the formation of a new nonfluorescent EthBr-DNA-complex system [63,65]. In the case of complexes **Ag1**–**3**, their addition to the EthBr-DNA solution causes the reduction in its emission intensity, indicating the interaction of complexes with DNA (as an illustration, the fluorescence emission spectra of EthBr–DNA system in the presence of an increasing concentration of **Ag2** complex were shown in Figure 7a). However, the calculated binding constants for all complexes (*K_A_*, Table 6) are significantly lower than that for EthBr (*K_A_* = 2 × 10^6^ M^−1^) [65], implying that the reason for the reduction in the emission intensity of the EthBr-DNA system in the presence of silver(I) complexes **Ag1**–**3** could be their binding to the EthBr-DNA and the formation of a new nonfluorescent EthBr-DNA-complex system.

The *K_sv_* values for the silver(I) complexes **Ag1**–**3** are low and suggest that they bind to ct-DNA through the non-intercalative (electrostatic) mode, which can be also concluded from the percentage of hypochromism up to 15%. For comparison, the percentage of hypochromism of 50% was previously obtained for lucigenin, which is proven as a DNA intercalator [72]. The values of *K_sv_* constants for the presently investigated complexes are slightly lower than those obtained for silver(I) complexes {[Ag(bpa)]NO_3_}*_n_* and {[Ag(bpa)_2_]CF_3_SO_3_·H_2_O}*_n_*, where bpa stands for 1,2-bis(4-pyridyl)ethane [62]. On the other hand, silver(I) complex [Ag(daf)(1,10-phen)]NO_3_ (daf = 4,5-diazafluoren-9-one and 1,10-phen = 1,10-phenanthroline) had high affinity for the intercalation between DNA base pairs, what can be concluded from the value of its *K_sv_* constant of 0.97 × 10^5^ M^−1^ at 37 °C [73]. From the values of *K_q_* constants higher than 2 × 10^10^ M^−1^·s^−1^, it can be seen that the mechanism of interaction between silver(I) complexes **Ag1**–**3** and DNA is a static quenching [65]. 

To further corroborate the possibility of complexes **Ag1**–**3** toward DNA interaction, gel electrophoresis methodology was also applied (Figure 7b). The slight reduction of the emission of the EthBr-DNA system was observable upon incubation with the tested complexes (Figure 7b), suggesting their weak interaction. The significant effect was only noticed for the **Ag3** complex at concentrations of 400 and 200 µM, as determined by Image J analysis (Figure S3).

## 3. Materials and Methods

### 3.1. Materials

All chemicals were of reagent-grade quality or higher and used without further purification. Solvents were used as received. The silver(I) salts (AgNO_3_, AgCF_3_SO_3,_ and AgPF_6_), thianthrene (tia), 1,8-naphthyridine (1,8-naph), ethanol, dichloromethane, acetonitrile, dimethyl sulfoxide (DMSO), deuterated dimethyl sulfoxide (DMSO-*d*_6_), acetonitrile (CD_3_CN), and chloroform (CDCl_3_), deuterium oxide (D_2_O), calf thymus DNA (ct-DNA), phosphate buffer saline (PBS), ethidium bromide (EthBr) and bovine serum albumin (BSA) were purchased from the Sigma-Aldrich (Munich, Germany).

### 3.2. Measurements

Elemental microanalyses of complexes **Ag1**–**3** for carbon, hydrogen, and nitrogen were done using a Perkin-Elmer 2400 Series II instrument (CHN) (Waltham, MA, USA). The ESI-HRMS spectra in the positive mode were recorded after dissolving 0.1 mg of silver(I) complexes **Ag1**–**3** in 1.0 mL acetonitrile with an Agilent 62224 accurate mass spectrometer (Santa Clara, CA, USA). The NMR (^1^H and ^13^C) spectra were recorded at room temperature on a Varian Gemini 2000 spectrometer (^1^H at 200 MHz, ^13^C at 50 MHz). Five milligrams of a compound were dissolved in 0.6 mL of CDCl_3_ (**Ag1**) and CD_3_CN/D_2_O (*v/v* 1:9) (**Ag2** and **Ag3**) and transferred into a 5 mm NMR tube. Chemical shifts were calibrated relative to those of the solvent. The abbreviations for the peak multiplicities are the follows *dd* (doublet of doublets) and *m* (multiplet). In order to investigate the stability of silver(I) complexes in solution, the ^1^H-NMR spectra were recorded immediately after their dissolution in DMSO-*d_6_*/D_2_O (*v/v* 1:9), as well as after 24 h and 48 h standing in the dark at room temperature. The IR spectra were recorded as KBr pellets on a Perkin-Elmer Spectrum 100 spectrometer (Shelton, CT, USA)) over the wavenumber range of 4000–450 cm^−1^. The UV-Vis spectra were recorded over the wavelength range of 900–200 nm on a Shimadzu UV-1800 spectrophotometer (Duisburg, Germany) after dissolving the silver(I) complexes in DMSO at room temperature, immediately and 24 h and 48 h after dissolution. The cyclic voltammetry (CV) measurements were performed using a potentiostat/galvanostat AutoLab PGSTAT204 (Utrecht, Netherlands). The cell (5.0 mL) consisted of a three-electrode system, a glassy carbon (GC) electrode as a working electrode, Ag/AgCl (saturated KCl) as a reference electrode, and a platinum wire as a counter electrode. All reported potentials are referred *versus* the Ag/AgCl (saturated KCl) reference electrode. The electrode surface was renewed before every measurement by polishing with Al_2_O_3_ micro-powder and with a piece of cotton due to the strong adsorption of the complexes. The concentration of the solutions of complexes **Ag1**–**3** in DMSO used for these measurements was 1 × 10^−3^ M. The emission spectra for DNA and BSA interactions of the complexes were recorded using Jasco FP-6600 spectrophotometer (Pfungstadt, Germany).

### 3.3. Synthesis of Silver(I) Complexes **Ag1**–**3**

Silver(I) complexes, [Ag(NO_3_)(tia)(H_2_O)]*_n_* (**Ag1**), [Ag(CF_3_SO_3_)(1,8-naph)]*_n_* (**Ag2**) and [Ag_2_(1,8-naph)_2_(H_2_O)_1.2_](PF_6_)_2_ (**Ag3**), were synthesized according to the modified procedure for the synthesis of silver(I) complexes with diazanaphthalenes [25] and their preliminary data have been presented [74,75]. The solution of 1.0 mmol of the corresponding silver(I) salt (169.9 mg of AgNO_3_ for **Ag1**, 256.9 mg of AgCF_3_SO_3_ for **Ag2,** and 252.8 mg of AgPF_6_ for **Ag3**) in 5.0 mL of ethanol was added slowly under stirring to the solution containing an equimolar amount of tia (216.3 mg) in 5.0 mL of dichloromethane for **Ag1** and 1,8-naph (130.2 mg) in 5.0 mL of ethanol for **Ag2** and **Ag3**. The reaction mixture was stirred for 3 h in the dark at room temperature, and then, the precipitate was filtered off and recrystallized in acetonitrile in the case of **Ag2** and **Ag3**. The obtained solutions were left at room temperature and after several days, colorless crystals of complexes **Ag1**–**3** were formed (**Ag1** complex crystallized from the mother solution). These crystals were filtered off and dried in the dark at ambient temperature. Yield (calculated on basis of the corresponding heterocyclic ligand): 315.3 mg (78%) for **Ag1**, 251.6 mg (65%) for **Ag2** and 267.8 mg (68%) for **Ag3**.

Anal. calcd for **Ag1** = C_12_H_10_AgNO_4_S_2_ (MW = 404.21): C, 35.66; H, 2.49; N, 3.47. Found: C, 35.39; H, 2.35; N, 3.29%. ESI-HRMS (CH_3_CN): *m/z* calcd for [Ag(tia)_2_]^+^: 540.5120; found: 540.9176; *m/z* calcd for [Ag(tia)(CH_3_CN)]^+^: 365.2420; found: 365.9370. ^1^H-NMR (200 MHz, CDCl_3_): *δ* = 7.23 (*m*, H2, H3, H7 and H8), 7.49 (*m*, H1, H4, H6 and H9) ppm. ^13^C-NMR (50 MHz, CDCl_3_): *δ* = 127.6 (C2, C3, C7 and C8), 128.6 (C1, C4, C6 and C9), 135.6 (C4a, C5a, C9a and C10a) ppm. IR (KBr, ν, cm^−1^): 3455 br (*ν*(O–H)), 3053 w (*ν*(C_ar_–H)), 1630 w, 1617 w, 1553 w, 1490 w, 1439 m, 1432 m (ν(C_ar_=C_ar_)), 1384 vs (ν_as_(NO_3_)), 761 m (γ(C_ar_–H)), 751 m (γ(C–S)). UV-Vis (DMSO, *λ*_max_, nm): 259 (*ε* = 6.7 × 10^4^ M^−1^·cm^−1^). 

Anal. calcd for **Ag2** = C_9_H_6_AgF_3_N_2_O_3_S (MW = 387.08): C, 27.93; H, 1.56; N, 7.24. Found: C, 28.00; H, 1.41; N, 7.23%. ESI-HRMS (CH_3_CN): *m/z* calcd for [Ag(1,8-naph)_2_]^+^: 367.0113; found: 367.0107; *m/z* calcd for [Ag(1,8-naph)(CH_3_CN)]^+^: 277.9847; found: 277.9843. ^1^H-NMR (200 MHz, D_2_O/CD_3_CN, *v/v* 1:9): *δ* 7.91 (*dd*, *J* = 8.2, 4.7 Hz, H4 and H5), 8.73 (*dd*, *J* = 8.3, 1.8 Hz, H3 and H6), 9.34 (*dd*, *J* = 4.7, 1.6 Hz, H2 and H7) ppm. ^13^C-NMR (50 MHz, D_2_O/CD_3_CN, *v/v* 1:9): *δ* = 124.3 (C4 and C5), 124.7 (C4a), 140.6 (C3 and C6), 155.9 (C2 and C7), 156.4 (C8a) ppm. IR (KBr, ν, cm^−1^): 3075 w (*ν*(C_ar_–H)), 1603 m, 1575 w, 1498 m, 146 w, 1457 w, 1441 w, 1411 w (ν(C_ar_=C_ar_) and ν(C_ar_=N)), 1254 vs (*ν*_as_(SO_3_)), 1163 s (*ν*_s_(CF_3_)), 1134 m (*ν*_as_(CF_3_)), 1044 m, 1032 s (*ν*_s_(SO_3_)), 833 m, 799 m, 636 m (γ(C_ar_–H)). UV-Vis (DMSO, *λ*_max_, nm): 307 (*ε* = 5.0 × 10^3^ M^−1^·cm^−1^). 

Anal. calcd for **Ag3** = C_16_H_14.4_Ag_2_F_12_N_4_O_1.2_P_2_ (MW = 787.58): C, 24.40; H, 1.84; N, 7.11. Found: C, 24.66; H, 1.69; N, 7.26%. ESI-HRMS (CH_3_CN): *m/z* calcd for [Ag(1,8-naph)_2_]^+^: 367.0113; found: 367.0107. ^1^H-NMR (200 MHz, D_2_O/CD_3_CN, *v/v* 1:9): *δ* 7.89 (*dd*, *J* = 8.2, 4.7 Hz, H4 and H5), 8.72 (*dd*, *J* = 8.2, 1.8 Hz, H3 and H6), 9.34 (*dd*, *J* = 4.7, 1.8 Hz, H2 and H7) ppm. ^13^C-NMR (50 MHz, D_2_O/CD_3_CN, *v/v* 1:9): *δ* = 124.6 (C4 and C5), 125.3 (C4a), 141.3 (C3 and C6), 152.8 (C2 and C7), 156.8 (C8a) ppm. IR (KBr, ν, cm^−1^): 3436 br (*ν*(O–H)), 3125 w, 3089 w (*ν*(C_ar_–H)), 1605 m, 1596 w, 1508 m, 1499 w, 1468 w, 1407 w (ν(C_ar_=C_ar_) and ν(C_ar_=N)), 831 vs (*ν*(PF_6_)), 847 vs, 799 s (γ(C_ar_–H)). UV-Vis (DMSO, *λ*_max_, nm): 307 (*ε* = 1.1 × 10^4^ M^−1^·cm^−1^). 

### 3.4. Air/Light Stability

The air/light stability of complexes **Ag1**–**3** and the silver(I) salts used for their synthesis (AgNO_3_, AgCF_3_SO_3,_ and AgPF_6_) were studied in direct light in an air atmosphere at room temperature [44]. For this purpose, sterile cellulose discs were impregnated with the corresponding silver(I) complex (5.0 μL of 50 mg/mL DMSO stock solution) and exposed to air and light. The stability was monitored visually over 48 h.

### 3.5. Crystallographic Data Collection and Refinement of the Structures

Single-crystal X-ray diffraction data were collected on an Agilent Technologies SuperNova Dual diffractometer (Yarnton, Oxfordshire, UK) with an Atlas detector using monochromated Mo-*K*α radiation (λ = 0.71073 Å) at 150 K. The data were processed using *CrysAlis Pro* [76]. The structure was solved by the SHELXT program [77] and refined by a full-matrix least-squares procedure based on *F^2^* with SHELXL [78] using the Olex2 program suite [79]. All non-hydrogen atoms were refined anisotropically. All the hydrogen atoms were readily located in difference Fourier maps and were subsequently treated as riding atoms in geometrically idealized positions with *U*_iso_(H) = *kU*_eq_(C,O), where *k* = 1.5 for OH groups and 1.2 for all other H atoms unless otherwise noted. In **Ag1**, the hydrogen atom H1A bonded to water molecule O1 was refined restraining the O–H bond length, while the hydrogen atom H1B was refined freely. In **Ag3**, the water molecule O1 was refined with a fixed 0.60 occupation factor. The crystallographic data are listed in Table S1.

### 3.6. Antimicrobial Studies

The minimum inhibitory concentration (MIC) values of silver(I) complexes **Ag1**–**3** alongside corresponding ligands, tia, and 1,8-naph, were determined according to the standard broth microdilution assays, recommended by the Standards of European Committee on Antimicrobial Susceptibility Testing (v 7.3.1: the method for the determination of broth dilution minimum inhibitory concentrations of antifungal agents for yeasts [80]) for *Candida* spp. (*C. albicans* ATCC 10,231, *C. parapsilosis* ATCC 22,019 and *C. krusei* ATCC 6258), and the National Committee for Clinical Laboratory Standards (M07-A8) for bacteria (*Pseudomonas aeruginosa* ATCC 10,332, *P. aeruginosa* BK25H [50], *Staphylococcus aureus* ATCC 25,923, *Listeria monocytogenes* NCTC 11,994 and *Escherichia coli* NCTC 9001). Stock solutions of silver(I) complexes were prepared in DMSO at a final concentration of 50 µg/mL and the highest tested concentration was 250 µg/mL (making sure that the final concentration of the DMSO in the assay was lower than 0.05%, *v/v*). The inoculums were 1 × 10^5^ colony forming units (cfu)/mL for *Candida* species, and 5 × 10^5^ cfu/mL for bacteria. The MIC value was recorded as the lowest concentration that inhibited the growth after 24 h at 37 °C, using the Tecan Infinite 200 Pro multiplate reader (Tecan Group Ltd., Männedorf, Switzerland).

### 3.7. Toxicity Assessment

#### 3.7.1. MTT Assay

Antiproliferative activity of silver(I) complexes **Ag1**–**3** was determined by 3-(4,5-dimethylthiazol-2-yl)-2,5-diphenyltetrazolium bromide (MTT) assay [81] on human lung fibroblasts cells (MRC-5, obtained from American Type Culture Collection (ATCC)). 1 × 10^4^ cells per well were cultured in the complete RPMI 1640 medium (Sigma-Aldrich, Munich, Germany) as a monolayer, and incubated with the silver(I) complexes, at a concentration from a maximum of 100 µg/mL, in a humidified atmosphere of 95% air and 5% CO_2_ at 37 °C for 48 h. The extent of MTT reduction was measured spectrophotometrically at 540 nm using Tecan Infinite 200 Pro multiplate reader (Tecan Group Ltd., Männedorf, Switzerland). Cytotoxicity was expressed as the concentration of the compound inhibiting cell growth by 50% (IC_50_) in comparison with the control (DMSO-treated cells).

#### 3.7.2. *C. elegans* Survival Assay

*Caenorhabditis elegans* N2 (*glp-4*; *sek-1*) was propagated under standard conditions, synchronized by hypochlorite bleaching, and cultured on nematode growth medium using *E. coli* OP50 as a food source, as described previously [82]. The *C. elegans* survival assay was carried out as described previously with some modifications [83,84]. In brief, synchronized worms (L4 stage) were suspended in a medium containing 95% M9 buffer (3.0 g of KH_2_PO_4_, 6.0 g of Na_2_HPO_4_, 5.0 g of NaCl, and 1 mL of 1 M MgSO_4_ × 7H_2_O in 1 L of water), 5% LB (Luria-Bertani) broth (Oxoid, Basingstoke, UK), and 10 μg of cholesterol (Sigma-Aldrich, Munich, Germany) per mL. The experiment was carried out in 96-well flat bottomed microtiter plates (Sarstedt, Nümbrecht, Germany) in the final volume of 100 μL per well. 25 μL of this suspension of nematodes (25–35 nematodes) were transferred to the wells of a 96-well microtiter plate, where 50 μL of the medium was previously added. Next, 25 μL of a solvent control (DMSO) or 25 μL of a concentrated solution was added to the test wells. Final concentrations of the complexes were 10, 5, and 1 µg/mL and 200, 100, and 50 µg/mL for ligands, made out from the stock solutions (50 mg/mL in DMSO) of each compound. Subsequently, the plates were incubated at 25 °C for 2 days. The fraction of dead worms was determined after 48 h by counting the number of dead worms and the total number of worms in each well, using a stereomicroscope (SMZ143-N2GG, Motic, Germany). The compounds were tested at least three times in each assay and each assay was repeated at least two times (n ≥ 6). As a negative control experiment, nematodes were exposed to the medium containing 1% DMSO.

### 3.8. BSA Binding Study

The BSA (16 μM) binding study was performed by carrying out tryptophan fluorescence quenching experiments in PBS solution (pH = 7.4). The quenching of the emission intensity of BSA at 366 nm was monitored using the increasing concentration of the complexes **Ag1**–**3** (0–310 μM). Fluorescence spectra were recorded in the range 280–500 nm with an excitation wavelength of 275 nm. The binding constants of the complexes (*K_A_*) and binding sites (*n*) were calculated using the following equation [63,85]:

log(F_0_ − F)/F = log*K_A_* + *n*log[complex] (1)

where *K_A_* is the binding constant of the silver(I) complex with BSA, while *n* represents the number of binding sites of the complex per BSA molecule.

### 3.9. Lipophilicity Assay

The lipophilicity of the silver(I) complexes **Ag1**–**3** was determined by the flask-shaking method [69]. The complexes were dissolved in DMSO, added to the water/*n*-octanol system, and vortexed for 1 h at room temperature. Thereafter, the solutions were allowed to stand for 24 h until the separation of the two phases was achieved. The concentration of complexes in both phases was determined by measuring absorbance values using previously determined calibration curves. *log*P values were calculated according to the equation:

*log*P = *log*(*c_0_*/*c_w_*) (2)

where *c_0_* and *c_w_* are the concentrations of the complex in the *n*-octanol and water phase, respectively.

### 3.10. DNA Interaction

#### 3.10.1. Fluorescence Emission Spectroscopy

The silver(I) complexes **Ag1**–**3** were dissolved in DMSO (10 mM). A stock solution of ct-DNA was prepared in PBS, and the concentration of this solution was determined from UV absorbance at 260 nm using the molar extinction coefficient *ε* = 6.6 × 10^3^ M^−1^·cm^−1^ [86]. A stock solution of ethidium bromide (EthBr) was prepared in DMSO (1.01 × 10^−2^ M) and kept at 4 °C prior to use. The competitive binding studies were carried out in PBS (pH = 7.4) by keeping [DNA]/[EthBr] = 10, while increasing the concentration of the complexes. Measurements were performed in the wavelength range of 525–800 nm with an excitation wavelength of 520 nm. The Stern–Volmer constants (*K_sv_*) were calculated using the following equation [63]:

F_0_/F = 1 + *K_q_τ_0_*[complex] = 1 + *K_sv_*[complex] (3)

where *F_0_* and *F* stand for the fluorescence intensity in the absence and presence of the silver(I) complex, respectively, *K_q_* represents the bimolecular quenching constant, and *τ_0_* (10^−8^ s) is the average fluorescence lifetime of the fluorophore in the absence of the quencher. The binding constants (*K_A_*) and apparent binding sites (*n*) were calculated as described above for BSA binding study [63,85].

#### 3.10.2. Gel Electrophoresis Assay

DNA interaction assay using gel electrophoresis was performed according to the previously published procedure [29] with commercial lambda bacteriophage DNA (300 ng, Thermo Scientific™). DNA solution of the final concentration 20 ng/μL was incubated with 400, 200 and 100 µM of silver(I) complexes **Ag1**–**3** and corresponding ligands, tia, and 1,8-naph, in 15 μL of reaction volume, for 1 h at 37 °C. 300 ng per lane of samples were run on 0.8% agarose gel with EthBr against a HyperLadder™ 1 kb DNA Ladder plus (FastGene) at 60 V for 1 h. Gels were visualized and analyzed using the Gel Doc EZ system (Bio-Rad, Life Sciences, Hercules, CA, USA), equipped with the Image Lab™ Software.

## 4. Conclusions

In this study, three new silver(I) complexes [Ag(NO_3_)(tia)(H_2_O)]*_n_* (**Ag1**), [Ag(CF_3_SO_3_)(1,8-naph)]*_n_* (**Ag2**) and [Ag_2_(1,8-naph)_2_(H_2_O)_1.2_](PF_6_)_2_ (**Ag3**), were synthesized, structurally characterized and biologically evaluated as potential antimicrobial agents. The present study confirms that the sulfur- and nitrogen-containing heterocycles, thianthrene and 1,8-naphthyridine, respectively, act as effective bridging ligands between two Ag(I) ions forming poly- or dinuclear complexes. The investigated complexes showed considerable activity against the Gram-positive *S. aureus*, two Gram-negative *P. aeruginosa* species and the tested *Candida* species, with low toxicity in vivo on the *C. elegans* nematode model. The type of bridging ligand in the investigated complexes plays an important role in determining their affinity toward DNA and BSA biomolecules. Thus, the **Ag1** complex with sulfur-containing heterocycle as bridging ligand has a higher binding affinity toward BSA in respect to **Ag2** and **Ag3** complexes having nitrogen-containing bridging ligand. On the other hand, the DNA interaction of the latter two complexes is more significant than with the **Ag1** complex. Nevertheless, it can be concluded that proteins could be more favorable binding sites for all three complexes in comparison to the nucleic acids. The obtained results from this study also suggest that the synthesized silver(I) complexes with thianthrene and 1,8-naphthyridine as ligands could be further evaluated as agents for the treatment of the mixed *Candida-Pseudomonas aeruginosa* and *Candida-Staphylococcus aureus* infections. 

## Figures and Tables

**Table 1:** Selected bond distances (Å) and bond angles (°) for complexes **Ag1**–**3**.

Distance (Å)	Ag1		Distance (Å)	Ag2	Ag3
Ag1–O1	2.360(2)		Ag1–O1	2.555(2)	2.449(4)
Ag1–O2	2.328(2)		Ag1···O2 ^ii^	2.645(2)	–
Ag1–S1	2.6198(7)		Ag1–N1	2.200(2)	2.186(3)
Ag1–S2 ^i^	2.6747(8)		Ag1–N2 ^i^	2.218(2)	2.184(3)
			Ag1···Ag1 ^i^	2.7871(5)	2.7235(6)
**Angle (°)**			**Angle (°)**		
O1–Ag1–O2	108.07(7)		O1–Ag1–N1	112.75(8)	91.94(14)
O1–Ag1–S1	105.18(5)		O1–Ag1–N2 ^i^	82.66(8)	96.78(14)
O2–Ag1–S1	124.24(5)		N1–Ag1–N2 ^i^	164.29(10)	169.76(12)
O1–Ag1–S2 ^i^	103.56(5)				
O2–Ag1–S2 ^i^	121.54(5)				
S1–Ag1–S2 ^i^	91.44(2)				

Symmetry codes for **Ag1**: (i) 1 + *x*, *y*, *z*; for **Ag2**: (i) 1 − *x*, 1 − *y*, 1 – *z*; (ii) 1 − *x*, 2 − *y*, 1 – *z*; for **Ag3**: (i) 1 − *x*, 1 − *y*, 2 − *z*.

**Table 2:** Hydrogen-bonding interactions in complexes **Ag1**–**3**.

D–H···A	D–H (Å)	H···A (Å)	D···A (Å)	D–H···A (^o^)
**Ag1**				
O1–H1A···O3 ^i^	0.820(18)	2.007(18)	2.825(3)	175(3)
O1–H1B···O4 ^ii^	0.82(3)	2.06(3)	2.849(3)	160(3)
C3–H3···O1 ^iii^	0.95	2.50	3.428(3)	166.6
C5–H5···O2 ^iv^	0.95	2.55	3.202(3)	125.6
C5–H5···O4 ^v^	0.95	2.51	3.279(4)	137.8
**Ag2**				
C1–H1···O2 ^ii^	0.95	2.43	3.169(4)	134
C7–H7···F2 ^iii^	0.95	2.46	3.301(4)	148
C8–H8···O3 ^iv^	0.95	2.40	3.299(4)	157
**Ag3**				
O1–H1A···F1	0.91	2.12	2.787(7)	129.0
O1–H1A···F1 ^ii^	0.91	2.11	2.810(7)	133.0
O1–H1B···F5	0.92	2.49	3.121(6)	126.6
O1–H1B···F5 ^iii^	0.92	2.51	3.084(6)	121.2
C1–H1···O1	0.95	2.45	3.152(6)	130.3
C8–H8···F5 ^iv^	0.95	2.52	3.141(5)	123.0

Symmetry codes for **Ag1**: (i) 1 − *x*, 1 − *y*, 1 − *z*; (ii) 2 − *x*, 1 − *y*, 1 − *z*; (iii) −1 + *x*, *y*, *z*; (iv) 1 − *x*, ½ + *y*, 3/2 − *z*; (v) 2 − *x*, ½ + *y*, 3/2 – *z*; for **Ag2**: (ii) 1 − *x*, 2 − *y*, 1 − *z*; (iii) −1 + *x*, −1 + *y*, *z*; (iv) *x*, −1 + *y*, *z*; for **Ag3**: (ii) 1 − *x*, 1 − *y*, 1 − *z*; (iii) 2 − *x*, 1 − *y*, 1 − *z*; (iv) −1 + *x*, *y*, 1 + *z*.

**Table 3:** Positions and redox reactions associated with observed cathodic and anodic peaks in cyclic voltammograms of complexes **Ag1**–**3** recorded at GC electrode in DMSO and 0.1 M TBAHP as a supporting electrolyte with a scan rate of 50 mV/s.

Silver(I) Complex	Oxidation Process (E, *V*)	Reduction Processes (E, *V*)	
	Ag(I)→Ag(II)	Ag(II)→Ag(I)	Ag(I)→Ag(0)
**Ag1**	+1.25	−0.43	−1.26
**Ag2**	+1.38	−0.27	−0.92
**Ag3**	+1.12	−0.19	-0.82

**Table 4:** Minimum inhibitory concentrations (MIC, µg/mL and µM) and cytotoxicity against healthy human fibroblasts MRC-5 (IC_50_, µg/mL and µM) of **Ag1**–**3** complexes and ligands against fungal and bacterial strains. Silver(I) complexes with 1,5-naphthyridine (1,5-naph) and silver(I) sulfadiazine (AgSD) were given for comparative purposes [30,31]. NT = Non tested. Reproduced with permissions from refs. [30,31]. Copyright (2019) Elsevier.

Compound Test		tia	Ag1	1,8-naph	Ag2	Ag3	1,5-naph	[Ag(NO_3_) (1,5-naph)]*_n_*	[Ag(CF_3_COO) (1,5-naph)]*_n_*	[Ag(CF_3_SO_3_) (1,5-naph)]*_n_*	AgSD
*C. albicans*	µg/mL	>200	7.81	>200	3.91	3.91	>250	3.1	3.1	1.25	3.6
	µM	>925	19.3	>1537	10.1	5.0	>1921	10.3	8.8	3.2	10
*C. krusei*	µg/mL	>200	1.56	>200	1.56	1.56	>250	0.78	1.56	1.25	0.89
	µM	>925	3.6	>1537	4.0	1.0	>1921	2.6	4.4	3.2	2.5
*C. parapsilosis*	µg/mL	>200	3.91	>200	3.91	7.81	>250	6.25	3.1	2.5	0.89
	µM	>925	9.7	>1537	10.1	9.9	>1921	20.8	8.8	6.5	2.5
*S. aureus*	µg/mL	>250	3.91	>250	7.81	7.81	>250	50	50	25	27
	µM	>1157	9.7	>1921	20.2	9.9	>1921	167	142	65	75
*L. monocytogenes*	µg/mL	>250	15.62	>250	15.62	125	>250	NT	NT	NT	NT
	µM	>1157	38.6	>1921	40.4	160	>1921	NT	NT	NT	NT
*E. coli*	µg/mL	>250	15.62	>250	31.25	15.62	>250	25	12.5	12.5	7.14
	µM	>1157	38.6	>1921	80.7	19.8	>1921	83	36	32	20
*P. aeruginosa* ATCC 10332	µg/mL	>200	6.25	>200	3.13	3.13	>250	25	25	25	8.93
	µM	>925	15.5	>1537	8.1	4.0	>1921	83	71	65	25
*P. aeruginosa* BK25H	µg/mL	>250	3.91	>250	3.91	3.91	>250	NT	NT	NT	NT
	µM	>1157	9.7	>1921	10.1	5.0	>1921	NT	NT	NT	NT
MRC-5 cells	µg/mL	>100	4.25	>100	3.75	3.65	>250	NT	NT	NT	3.6
	µM	>462	10.5	>768	9.7	4.6	>1921	NT	NT	NT	10

**Table 5:** Values of the binding constants of complexes **Ag1**–**3** with BSA.

Complex	*K_sv_* (M^−1^)	Hypochromism (%)	*K_q_* (M^−1^·s^−1^)	*K_A_* (M^−1^)	*n*
**Ag1**	(1.56 ± 0.01) × 10^5^	78.0	1.56 × 10^13^	3.05 × 10^6^	1.41
**Ag2**	(1.34 ± 0.01) × 10^4^	70.2	1.34 × 10^12^	2.88 × 10^4^	1.12
**Ag3**	(2.70 ± 0.05) × 10^4^	68.7	2.70 × 10^12^	6.08 × 10^5^	1.37

**Table 6:** Values of the binding constants of complexes **Ag1**–**Ag3** with ct-DNA.

Complex	*K_sv_* (M^−1^)	Hypochromism (%)	*K_q_* (M^−1^·s^−1^)	*K_A_* (M^−1^)	*n*
**Ag1**	(7.33 ± 0.33) × 10^2^	12.7	7.33 × 10^10^	1.05 × 10^2^	0.76
**Ag2**	(1.29 ± 0.03) × 10^3^	15.2	1.29 × 10^11^	4.83 × 10^2^	0.90
**Ag3**	(1.15 ± 0.07) × 10^3^	15.1	1.15 × 10^11^	4.33 × 10^2^	0.88

## Data Availability

The experimental data used to support the findings of this study are available on request from the corresponding author.

## Supplementary Materials

The following are available online. CCDC 2065519–2065521 contains the supplementary crystallographic data for this paper. These data can be obtained free of charge via www.ccdc.cam.ac.uk/data_request/cif or by emailing data_request@ccdc.cam.ac.uk or by contacting The Cambridge Crystallography Data Centre, 12 Union Road, Cambridge CB2 1EZ, UK; fax: +44-1223-336033. ^1^H and ^13^C-NMR spectra for **Ag1**–**3**, thianthrene (tia) and 1,8-naphthyridine (1,8-naph). Experimental data for thianthrene (tia) and 1,8-naphthyridine (1,8-naph). Figure S1: Time stability of **Ag3** complex followed by UV-Vis spectrophotometry at room temperature in DMSO, Figure S2: Air/light stability of silver(I) complexes **Ag1**–**3** and corresponding silver(I) salts used for their synthesis, Figure S3: Quantification of the interaction of silver(I) complexes **Ag1**–**3** with commercial lambda bacteriophage DNA by gel electrophoresis done in the Excel program. ImageJ program was used for figure analysis, Table S1: Details of the crystal structure determination for complexes **Ag1**–**3**.

## Sample Availability

Samples of the compounds **Ag1**–**3** are available from the authors.

## References

[1] Tacconelli E., Pezzani M.D. (2019). Public health burden of antimicrobial resistance in Europe. Lancet Infect. Dis..

[2] O’Neill J. (2014). Review on Antimicrobial Resistance Antimicrobial Resistance: Tackling a Crisis for the Health and Wealth of Nations.

[3] Barillo D.J., Marx D.E. (2014). Silver in medicine: A brief history BC 335 to present. Burns.

[4] Dai T., Huang Y.-Y., Sharma S.K., Hashmi J.T., Kurup D.B., Hamblin M.R. (2010). Topical antimicrobials for burn wound infections. Recent Pat. Antiinfect. Drug Discov..

[5] Nunes J.H.B., Bergamini F.R.G., Lustri W.R., de Paiva P.P., Ruiz A.L.T.G., de Carvalho J.E., Corbi P.P. (2017). Synthesis, characterization and in vitro biological assays of a silver(I) complex with 5-fluorouracil: A strategy to overcome multidrug resistant tumor cells. J. Fluor. Chem..

[6] Cavicchioli M., Massabni A.C., Heinrich T.A., Costa-Neto C.M., Abrão E.P., Fonseca B.A.L., Castellano E.E., Corbi P.P., Lustri W.R., Leite C.Q.F. (2010). Pt(II) and Ag(I) complexes with acesulfame: Crystal structure and a study of their antitumoral, antimicrobial and antiviral activities. J. Inorg. Biochem..

[7] Zachariadis P.C., Hadjikakou S.K., Hadjiliadis N., Skoulika S., Michaelides A., Balzarini J., De Clercq E. (2004). Synthesis, characterization and in vitro study of the cytostatic and antiviral activity of new polymeric silver(I) complexes with ribbon structures derived from the conjugated heterocyclic thioamide 2-mercapto-3,4,5,6-tetra- hydropyrimidine. Eur. J. Inorg. Chem..

[8] Lansdown A.B.G. (2006). Silver in health care: Antimicrobial effects and safety in use. Curr. Probl. Dermatol..

[9] Rizzello L., Pompa P.P. (2014). Nanosilver-based antibacterial drugs and devices: Mechanisms, methodological drawbacks, and guidelines. Chem. Soc. Rev..

[10] Medici S., Peana M., Nurchi V.M., Zoroddu M.A. (2019). Medical uses of silver: History, myths, and scientific evidence. J. Med. Chem..

[11] Eckhardt S., Brunetto P.S., Gagnon J., Priebe M., Giese B., Fromm K.M. (2013). Nanobio silver: Its interactions with peptides and bacteria, and its uses in medicine. Chem. Rev..

[12] Nunes J.H.B., de Paiva R.E.F., Cuin A., Lustri W.R., Corbi P.P. (2015). Silver complexes with sulfathiazole and sulfamethoxazole: Synthesis, spectroscopic characterization, crystal structure and antibacterial assays. Polyhedron.

[13] Tomašić T., Mašič L.P. (2009). Rhodanine as a privileged scaffold in drug discovery. Curr. Med. Chem..

[14] Matysiak J. (2015). Biological and pharmacological activities of 1,3,4-thiadiazole based compounds. Mini Rev. Med. Chem..

[15] Yadav G., Ganguly S. (2015). Structure activity relationship (SAR) study of benzimidazole scaffold for different biological activities: A mini-review. Eur. J. Med. Chem..

[16] Pathania S., Narang R.K., Rawal R.K. (2019). Role of sulphur-heterocycles in medicinal chemistry: An update. Eur. J. Med. Chem..

[17] Kerru N., Gummidi L., Maddila S., Gangu K.K., Jonnalagadda S.B. (2020). A review on recent advances in nitrogen-containing molecules and their biological applications. Molecules.

[18] Kim D.E., Kim Y., Cho D.-H., Jeong S.-Y., Kim S.-B., Suh N., Lee J.S., Choi E.K., Koh J.-Y., Hwang J.J. (2015). Raloxifene induces autophagy-dependent cell death in breast cancer cells via the activation of AMP-activated protein kinase. Mol. Cells.

[19] Herdeiro M.T., Soares S., Silva T., Roque F., Figueiras A. (2016). Impact of rosiglitazone safety alerts on oral antidiabetic sales trends: A countrywide study in Portugal. Fundam. Clin. Pharmacol..

[20] Séïde M., Marion M., Mateescu M.A., Averill-Bates D.A. (2016). The fungicide thiabendazole causes apoptosis in rat hepatocytes. Toxicol. In Vitro.

[21] Zhang L., Peng X.-M., Damu G.L.V., Geng R.-X., Zhou C.-H. (2013). Comprehensive review in current developments of imidazole-based medicinal chemistry. Med. Res. Rev..

[22] Jain S., Chandra V., Jain P.K., Pathak K., Pathak D., Vaidya A. (2019). Comprehensive review on current developments of quinoline-based anticancer agents. Arabian J. Chem..

[23] Savić N.D., Glišić B.Đ., Wadepohl H., Pavic A., Senerovic L., Nikodinovic-Runic J., Djuran M.I. (2016). Silver(I) complexes with quinazoline and phthalazine: Synthesis, structural characterization and evaluation of biological activities. MedChemComm.

[24] Savić N.D., Milivojevic D.R., Glišić B.Đ., Ilic-Tomic T., Veselinovic J., Pavic A., Vasiljevic B., Nikodinovic-Runic J., Djuran M.I. (2016). A comparative antimicrobial and toxicological study of gold(III) and silver(I) complexes with aromatic nitrogen-containing heterocycles: Synergistic activity and improved selectivity index of Au(III)/Ag(I) complexes mixture. RSC Adv..

[25] Glišić B.Đ., Senerovic L., Comba P., Wadepohl H., Veselinovic A., Milivojevic D.R., Djuran M.I., Nikodinovic-Runic J. (2016). Silver(I) complexes with phthalazine and quinazoline as effective agents against pathogenic *Pseudomonas aeruginosa* strains. J. Inorg. Biochem..

[26] Coyle B., Kavanagh K., McCann M., Devereux M., Geraghty M. (2003). Mode of anti-fungal activity of 1,10-phenanthroline and its Cu(II), Mn(II) and Ag(I) complexes. Biometals.

[27] McCann M., Coyle B., McKay S., McCormack P., Kavanagh K., Devereux M., McKee V., Kinsella P., O’Connor R., Clynes M. (2004). Synthesis and X-ray crystal structure of [Ag(phendio)_2_]ClO_4_ (phendio = 1,10-phenanthroline-5,6-dione) and its effects on fungal and mammalian cells. Biometals.

[28] Rowan R., Moran C., McCann M., Kavanagh K. (2009). Use of *Galleria mellonella* larvae to evaluate the in vivo anti-fungal activity of [Ag_2_(mal)(phen)_3_]. Biometals.

[29] Savić N.D., Vojnovic S., Glišić B.Đ., Crochet A., Pavic A., Janjić G.V., Pekmezović M., Opsenica I.M., Fromm K.M., Nikodinovic-Runic J. (2018). Mononuclear silver(I) complexes with 1,7-phenanthroline as potent inhibitors of *Candida* growth. Eur. J. Med. Chem..

[30] Pavic A., Savić N.D., Glišić B.Đ., Crochet A., Vojnovic S., Kurutos A., Stanković D.M., Fromm K.M., Nikodinovic-Runic J., Djuran M.I. (2019). Silver(I) complexes with 4,7-phenanthroline efficient in rescuing the zebrafish embryos of lethal *Candida albicans* infection. J. Inorg. Biochem..

[31] Đurić S., Vojnovic S., Pavic A., Mojicevic M., Wadepohl H., Savić N.D., Popsavin M., Nikodinovic-Runic J., Djuran M.I., Glišić B.Đ. (2020). New polynuclear 1,5-naphthyridine-silver(I) complexes as potential antimicrobial agents: The key role of the nature of donor coordinated to the metal center. J. Inorg. Biochem..

[32] Aslanidis P., Hatzidimitriou A.G., Andreadou E.G., Pantazaki A.A., Voulgarakis N. (2015). Silver(I) complexes of N-methylbenzothiazole-2-thione: Synthesis, structures and antibacterial activity. Mat. Sci. Eng. C.

[33] Aulakh J.K., Lobana T.S., Sood H., Arora D.S., Kaur R., Singh J., Garcia-Santos I., Kaure M., Jasinski J.P. (2019). Silver derivatives of multi-donor heterocyclic thioamides as antimicrobial/anticancer agents: Unusual bio-activity against methicillin resistant *S. aureus*, *S. epidermidis*, and *E. faecalis* and human bone cancer MG63 cell line. RSC Adv..

[34] Leung M.C.K., Williams P.L., Benedetto A., Au C., Helmcke K.J., Aschner M., Meyer J.N. (2008). *Caenorhabditis elegans*: An emerging model in biomedical and environmental toxicology. Toxicol. Sci..

[35] Potapov A.S., Nudnova E.A., Khlebnikov A.I., Ogorodnikov V.D., Petrenko T.V. (2015). Synthesis, crystal structure and electrocatalytic activity of discrete and polymeric copper(II) complexes with bitopic bis(pyrazol-1-yl)methane ligands. Inorg. Chem. Commun..

[36] Johnston D.H., Shriver D.F. (1993). Vibrational study of the trifluoromethanesulfonate anion: Unambiguous assignment of the asymmetric stretching modes. Inorg. Chem..

[37] Van Albada G.A., Smeets W.J.J., Spek A.L., Reedijk J. (1997). Synthesis, spectroscopic properties and X-ray crystal structures of two dinuclear alkoxo-bridged copper(II) compounds with the ligand bis(1-methyl-2-benzimidazolyl) propane. A unique alkoxo-bridged Cu(II) dinuclear compound with an additional bidentate bridging triflate anion. Inorg. Chim. Acta.

[38] El Hamdani H., El Amane M., Duhayon C. (2018). Studies on the syntheses, structural characterization, antimicrobial of the co-crystal 1,10-phenanthrolin-1-ium(1,10-phenH^+^)-caffeine(caf)-hexafluorophosphate. J. Mol. Struct..

[39] Nakajima Y., Shiraishi Y., Tsuchimoto T., Ozawa F. (2011). Synthesis and coordination behavior of Cu^I^ bis(phosphaethenyl)pyridine complexes. Chem. Commun..

[40] Munakata M., Yan S.G., Ino I., Kuroda-Sowa T., Maekawa M., Suenaga Y. (1998). Synthesis and structure of a novel bis(μ-η^2^-thianthrene )disilver(I) bis(perchlorate). Inorg. Chim. Acta.

[41] Glišić B.Đ., Warżajtis B., Hoffmann M., Rychlewska U., Djuran M.I. (2020). Mononuclear gold(III) complexes with diazanaphthalenes: The influence of the position of nitrogen atoms in the aromatic rings on the complex crystalline properties. RSC Adv..

[42] Zhang J.-A., Pan M., Zhang J.-Y., Zhang H.-K., Fan Z.-J., Kang B.-S., Su C.-Y. (2009). Syntheses, structures and bioactivities of silver(I) complexes with a tridentate heterocyclic N- and S-ligand. Polyhedron.

[43] Jiang Y., Zhu C.-F., Zheng Z., He J.-B., Wang Y. (2016). Synthesis, characterization and antibacterial activity of a biocompatible silver complex based on 2,2′-bipyridine and 5-sulfoisophthalate. Inorg. Chim. Acta.

[44] Kalinowska-Lis U., Felczak A., Chęcińska L., Zawadzka K., Patyna E., Lisowska K., Ochocki J. (2015). Synthesis, characterization and antimicrobial activity of water-soluble silver(I) complexes of metronidazole drug and selected counter-ions. Dalton Trans..

[45] Cardoso J.M.S., Correia I., Galvão A.M., Marques F., Carvalho M.F.N.N. (2017). Synthesis of Ag(I) camphor sulphonylimine complexes and assessment of their cytotoxic properties against *cisplatin*-resistant A2780cisR and A2780 cell lines. J. Inorg. Biochem..

[46] Sirajuddin M., Ali S., Badshah A. (2013). Drug–DNA interactions and their study by UV–Visible, fluorescence spectroscopies and cyclic voltametry. J. Photochem. Photobiol. B Biol..

[47] Wu J.-Y., Pan Y.-L., Zhang X.-J., Sun T., Tian Y.-P., Yang J.-X., Chen Z.-N. (2007). Synthesis, photoluminescence and electrochemical properties of a series of carbazole-functionalized ligands and their silver(I) complexes. Inorg. Chim. Acta.

[48] Połczyński P., Jurczakowski R., Grochala W. (2013). Strong and long-lived free-radical oxidizer based on silver(II). Mechanism of Ag(I) electrooxidation in concentrated H_2_SO_4_. J. Phys. Chem. C.

[49] Andrejević T.P., Milivojevic D., Glišić B.Đ., Kljun J., Stevanović N.L., Vojnovic S., Medic S., Nikodinovic-Runic J., Turel I., Djuran M.I. (2020). Silver(I) complexes with different pyridine-4,5-dicarboxylate ligands as efficient agents for the control of cow mastitis associated pathogens. Dalton Trans..

[50] Milivojevic D., Šumonja N., Medić S., Pavic A., Moric I., Vasiljevic B., Senerovic L., Nikodinovic-Runic J. (2018). Biofilm-forming ability and infection potential of *Pseudomonas aeruginosa* strains isolated from animals and humans. Pathog. Dis..

[51] Banti C.N., Hadjikakou S.K. (2013). Anti-proliferative and anti-tumor activity of silver(I) compounds. Metallomics.

[52] Dakal T.C., Kumar A., Majumdar R.S., Yadav V. (2016). Mechanistic basis of antimicrobial actions of silver nanoparticles. Front. Microbiol..

[53] Ortego L., Gonzalo-Asensio J., Laguna A., Villacampa M.D., Gimeno M.C. (2015). (Aminophosphane)gold(I) and silver(I) complexes as antibacterial agents. J. Inorg. Biochem..

[54] Francius G., Domenech O., Mingeot-Leclercq M.P., Dufrêne Y.F. (2008). Direct observation of *Staphylococcus aureus* cell wall digestion by lysostaphin. J. Bacteriol..

[55] Xiong H., Pears C., Woollard A. (2017). An enhanced *C. elegans* based platform for toxicity assessment. Sci. Rep..

[56] Hunt P.R. (2017). The *C. elegans* model in toxicity testing. J. Appl. Toxicol..

[57] Shi J.-H., Pan D.-Q., Jiang M., Liu T.-T., Wang Q. (2017). In vitro study on binding interaction of quinapril with bovine serum albumin (BSA) using multi-spectroscopic and molecular docking methods. J. Biomol. Struct. Dyn..

[58] Shi J.-H., Zhu Y.-Y., Wang J., Chen J., Shen Y.-J. (2013). Intermolecular interaction of prednisolone with bovine serum albumin: Spectroscopic and molecular docking methods. Spectrochim. Acta A Mol. Biomol. Spectrosc..

[59] Anbazhagan V., Renganathan R. (2008). Study on the binding of 2,3-diazabicyclo [2.2.2]oct-2-ene with bovine serum albumin by fluorescence spectroscopy. J. Lumin..

[60] Li Z., Niu M., Chang G., Zhao G.C. (2015). Chiral manganese(IV) complexes derived from Schiff base ligands: Synthesis, characterization, in vitro cytotoxicity and DNA/BSA interaction. J. Photochem. Photobiol. B Biol..

[61] Bhat S.S., Kumbhar A.A., Heptullah H., Khan A.A., Gobre V.V., Gejji S.P., Puranik V.G. (2011). Synthesis, electronic structure, DNA and protein binding, DNA cleavage, and anticancer activity of fluorophore-labeled copper(II) complexes. Inorg. Chem..

[62] Đurić S.Ž., Vojnovic S., Andrejević T.P., Stevanović N.L., Savić N.D., Nikodinovic-Runic J., Glišić B.Đ., Djuran M.I. (2020). Antimicrobial activity and DNA/BSA binding affinity of polynuclear silver(I) complexes with 1,2-bis(4-pyridyl)ethane/ethene as bridging ligands. Bioinorg. Chem. Appl..

[63] Smolénski P., Pettinari C., Marchetti F., Guedes da Silva M.F.C., Lupidi G., Patzmay G.V.B., Petrelli D., Vitali L.A., Pomberio A.J.L. (2015). Syntheses, structures, and antimicrobial activity of new remarkably light-stable and water-soluble tris(pyrazolyl)methanesulfonate silver(I) derivatives of *N*-methyl-1,3,5-triaza-7-phosphaadamantane salt—[mPTA]BF_4_. Inorg. Chem..

[64] Timerbaev A.R., Hartinger C.G., Aleksenko S.S., Keppler B.K. (2006). Interactions of antitumor metallodrugs with serum proteins:  advances in characterization using modern analytical methodology. Chem. Rev..

[65] Shi Y., Guo C., Sun Y., Liu Z., Xu F., Zhang Y., Wen Z., Li Z. (2011). Interaction between DNA and microcystin-LR studied by spectra analysis and atomic force microscopy. Biomacromolecules.

[66] Rajendiran V., Karthik R., Palaniandavar M., Stoeckli-Evans H., Periasamy V.S., Akbarsha M.A., Srinag B.S., Krishnamurthy H. (2007). Mixed-ligand copper(II)-phenolate complexes:  effect of coligand on enhanced DNA and protein binding, DNA cleavage, and anticancer activity. Inorg. Chem..

[67] Fetzer L., Boff B., Ali M., Xiangjun M., Collin J.-P., Sirlin C., Gaiddon C., Pfeffer M. (2011). Library of second-generation cycloruthenated compounds and evaluation of their biological properties as potential anticancer drugs: Passing the nanomolar barrier. Dalton Trans..

[68] Li J.J., Tian M., Tian Z., Zhang S., Yan C., Shao C., Liu Z. (2018). Half-sandwich iridium(III) and ruthenium(II) complexes containing P^P-chelating ligands: A new class of potent anticancer agents with unusual redox features. Inorg. Chem..

[69] Puckett C.A., Barton J.K. (2007). Methods to explore cellular uptake of ruthenium complexes. J. Am. Chem. Soc..

[70] Ghose A.K., Viswanadhan V.N., Wendoloski J.J. (1999). A knowledge-based approach in designing combinatorial or medicinal chemistry libraries for drug discovery. 1. A qualitative and quantitative characterization of known drug databases. J. Comb. Chem..

[71] Turel I., Kljun J. (2011). Interactions of metal ions with DNA, its constituents and derivatives, which may be relevant for anticancer research. Curr. Top. Med. Chem..

[72] Wu H.-L., Li W.-Y., He X.-W., Miao K., Liang H. (2002). Spectral studies of the binding of lucigenin, a bisacridinium derivative, with double-helix DNA. Anal. Bioanal. Chem..

[73] Movahedi E., Rezvani A.R., Razmazma H. (2019). Binding interaction of a heteroleptic silver(I) complex with DNA: A joint experimental and computational study. Int. J. Biol. Macromol..

[74] Ašanin D.P., Andrejević T.P., Skaro-Bogojevic S., Stevanović N.L., Aleksic I., Milivojevic D., Perdih F., Turel I., Djuran M.I., Glišić B.Đ. (2020). Polynuclear silver(I) complex with thianthrene: Structural characterization, antimicrobial activity and interaction with biomolecules. Proceedings.

[75] Ašanin D.P., Andrejević T.P., Skaro-Bogojevic S., Perdih F., Turel I., Nikodinovic-Runic J., Djuran M.I., Glišić B.Đ. Antimicrobial activity and DNA/BSA binding study of new silver(I) complexes with 1,8-naphthyridine. Proceedings of the 6th International Electronic Conference on Medicinal Chemistry, Session General: Presentations.

[76] Agilent Technologies Ltd. (2013). CrysAlisPro, Version 1.171.39.46.

[77] Sheldrick G.M. (2015). SHELXT—Integrated space-group and crystal-structure determination. Acta Cryst..

[78] Sheldrick G.M. (2015). Crystal structure refinement with SHELXL. Acta Cryst..

[79] Dolomanov O.V., Bourhis L.J., Gildea R.J., Howard J.A.K., Puschmann H. (2009). OLEX2: A complete structure solution, refinement and analysis program. J. Appl. Cryst..

[80] Arendrup M.C., Cuenca-Estrella M., Lass-Flörl C., Hope W. (2012). EUCAST-AFST. EUCAST technical note on the EUCAST definitive document EDef 7.2: Method for the determination of broth dilution minimum inhibitory concentrations of antifungal agents for yeasts EDef 7.2 (EUCAST-AFST). Clin. Microbiol. Infect..

[81] Hansen M.B., Nielsen S.E., Berg K. (1989). Re-examination and further development of a precise and rapid dye method for measuring cell growth/cell kill. J. Immunol. Methods.

[82] Stiernagle T. (2006). Maintenance of *C. elegans*. WormBook.

[83] Scoffone V.C., Chiarelli L.R., Makarov V., Brackman G., Israyilova A., Azzalin A., Forneris F., Riabova O., Savina S., Coenye T. (2016). Discovery of new diketopiperazines inhibiting *Burkholderia cenocepacia* quorum sensing in vitro and in vivo. Sci. Rep..

[84] Brackman G., Cos P., Maes L., Nelis H.J., Coenye T. (2011). Quorum sensing inhibitors increase the susceptibility of bacterial biofilms to antibiotics in vitro and in vivo. Antimicrob. Agents Chemother..

[85] Wolfe A., Shimer G.H., Meehan T. (1987). Polycyclic aromatic hydrocarbons physically intercalate into duplex regions of denatured DNA. Biochemistry.

[86] Bera R., Sahoo B.K., Ghosh K.S., Dasgupta S. (2008). Studies on the interaction of isoxazolcurcumin with calf thymus DNA. Int. J. Biol. Macromol..

