# Supplementary material for: Structural Characterization, Antimicrobial Activity and BSA/DNA Binding Affinity of New Silver(I) Complexes with Thianthrene and 1,8-Naphthyridine
Source: Molecules. 2021 Mar 26;26(7):1871. doi: 10.3390/molecules26071871 (PMC8037121; doi:10.3390/molecules26071871)

checkCIF/PLATON report

Structure factors have been supplied for datablock(s) I

THIS REPORT IS FOR GUIDANCE ONLY. IF USED AS PART OF A REVIEW PROCEDURE FOR PUBLICATION, IT SHOULD NOT REPLACE THE EXPERTISE OF AN EXPERIENCED CRYSTALLOGRAPHIC REFEREE.

No syntax errors found. CIF dictionary Interpreting this report

Datablock: I

```
Bond precision:  C-C = 0.0047 A          Wavelength=0.71073

Cell:            a=8.5216(4)     b=9.1114(5)         c=15.2361(8)
                 alpha=90        beta=102.225(5)     gamma=90
Temperature:     150 K

                Calculated                 Reported
Volume          1156.16(11)                1156.16(11)
Space group     P 21/c                     P 21/c
Hall group      -P 2ybc                    -P 2ybc
Moiety formula  C9 H6 Ag F3 N2 O3 S        C9 H6 Ag F3 N2 O3 S
Sum formula     C9 H6 Ag F3 N2 O3 S        C9 H6 Ag F3 N2 O3 S
Mr              387.09                     387.09
Dx,g cm-3       2.224                      2.224
Z               4                          4
Mu (mm-1)       1.969                      1.969
F000            752.0                      752.0
F000'           749.33
h,k,lmax        11,11,19                   11,11,19
Nref            2643                       2642
Tmin,Tmax       0.630,0.674                0.623,1.000
Tmin'           0.304

Correction method= # Reported T Limits: Tmin=0.623 Tmax=1.000
AbsCorr = MULTI-SCAN

Data completeness= 1.000          Theta(max)= 27.480

R(reflections)= 0.0294( 2296)     wR2(reflections)= 0.0762( 2642)

S = 1.101                  Npar= 172
```

The following ALERTS were generated. Each ALERT has the format
test-name_ALERT_alert-type_alert-level.
Click on the hyperlinks for more details of the test.

Alert level C

```
PLAT242_ALERT_2_C Low    'MainMol' Ueq as Compared to Neighbors of         C9 Check
```

Alert level G

```
PLAT004_ALERT_5_G Polymeric Structure Found with Maximum Dimension          1 Info
PLAT434_ALERT_2_G Short Inter HL..HL Contact F1       ..F3               2.78 Ang.
                                           1-x,-1/2+y,1/2-z  =      2_645 Check
PLAT910_ALERT_3_G Missing # of FCF Reflection(s) Below Theta(Min).          1 Note
PLAT941_ALERT_3_G Average HKL Measurement Multiplicity ...........        2.4 Low
PLAT978_ALERT_2_G Number C-C Bonds with Positive Residual Density.          0 Info
```

0 **ALERT level A** = Most likely a serious problem - resolve or explain
0 **ALERT level B** = A potentially serious problem, consider carefully
1 **ALERT level C** = Check. Ensure it is not caused by an omission or oversight
5 **ALERT level G** = General information/check it is not something unexpected

0 ALERT type 1 CIF construction/syntax error, inconsistent or missing data
3 ALERT type 2 Indicator that the structure model may be wrong or deficient
2 ALERT type 3 Indicator that the structure quality may be low
0 ALERT type 4 Improvement, methodology, query or suggestion
1 ALERT type 5 Informative message, check

checkCIF publication errors

Alert level A

```
PUBL012_ALERT_1_A  _publ_section_abstract is missing.
            Abstract of paper in English.
PUBL024_ALERT_1_A The number of authors is greater than 9.
              Please specify the role of each of the co-authors
              for your paper.
```

Alert level G

```
PUBL017_ALERT_1_G The _publ_section_references section is missing or
            empty.
```

2 **ALERT level A** = Data missing that is essential or data in wrong format
1 **ALERT level G** = General alerts. Data that may be required is missing

Publication of your CIF

You should attempt to resolve as many as possible of the alerts in all categories. Often the minor alerts point to easily fixed oversights, errors and omissions in your CIF or refinement strategy, so attention to these fine details can be worthwhile. In order to resolve some of the more serious problems it may be necessary to carry out additional measurements or structure refinements. However, the nature of your study may justify the reported deviations from journal submission requirements and the more serious of these should be commented upon in the discussion or experimental section of a paper or in the "special_details" fields of the CIF. *checkCIF* was carefully designed to identify outliers and unusual parameters, but every test has its limitations and alerts that are not important in a particular case may appear. Conversely, the absence of alerts does not guarantee there are no aspects of the results needing attention. It is up to the individual to critically assess their own results and, if necessary, seek expert advice.

If level A alerts remain, which you believe to be justified deviations, and you intend to submit this CIF for publication in a journal, you should additionally insert an explanation in your CIF using the Validation Reply Form (VRF) below. This will allow your explanation to be considered as part of the review process.

Validation response form

Please find below a validation response form (VRF) that can be filled in and pasted into your CIF.

```
# start Validation Reply Form
_vrf_PUBL012_GLOBAL
;
PROBLEM: _publ_section_abstract is missing.
RESPONSE: ...
;
_vrf_PUBL024_GLOBAL
;
PROBLEM: The number of authors is greater than 9.
RESPONSE: ...
;
# end Validation Reply Form
```

If you wish to submit your CIF for publication in Acta Crystallographica Section C or E, you should upload your CIF via the web. If you wish to submit your CIF for publication in IUCrData you should upload your CIF via the web. If your CIF is to form part of a submission to another IUCr journal, you will be asked, either during electronic submission or by the Co-editor handling your paper, to upload your CIF via our web site.

PLATON version of 05/12/2020; check.def file version of 05/12/2020

Datablock I - ellipsoid plot

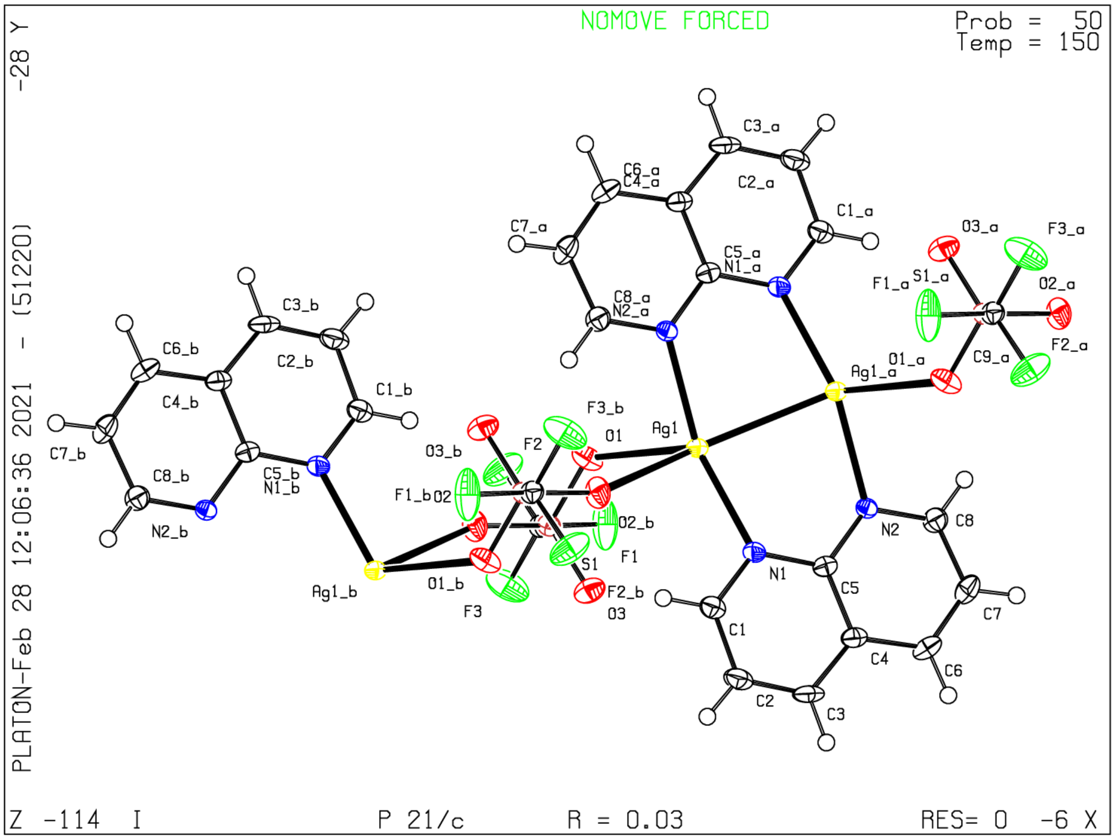

Supplement: Supplementary file 1 [file molecules-26-01871-s001.zip › molecules-1157613-final-SM/Ag2-checkcif.pdf]
