# Supplementary material for: Structural Characterization, Antimicrobial Activity and BSA/DNA Binding Affinity of New Silver(I) Complexes with Thianthrene and 1,8-Naphthyridine
Source: Molecules. 2021 Mar 26;26(7):1871. doi: 10.3390/molecules26071871 (PMC8037121; doi:10.3390/molecules26071871)
Supplement: Supplementary file 1 [file molecules-26-01871-s001.zip › molecules-1157613-final-SM/Supplementary Materials_revised.docx]

**Structural characterization, antimicrobial activity and BSA/DNA binding affinity of new silver(I) complexes with thianthrene and 1,8-naphthyridine**

Darko P. Ašanin ^1^, Sanja Skaro Bogojevic ^2^, Franc Perdih ^3^, Tina P. Andrejević ^4^, Dusan Milivojevic ^2^, Ivana Aleksic ^2^, Jasmina Nikodinovic-Runic ^2,*^, Biljana Đ. Glišić ^4,*^, Iztok Turel ^3,*^ and Miloš I. Djuran^5,*^

^1^ University of Kragujevac, Institute for Information Technologies Kragujevac, Department of Science, Jovana Cvijića bb, 34000 Kragujevac, Serbia; [darko.asanin@uni.kg.ac.rs](mailto:darko.asanin@uni.kg.ac.rs) (D.A.)

^2^ University of Belgrade, Institute of Molecular Genetics and Genetic Engineering, Vojvode Stepe 444a, 11042 Belgrade, Serbia; [sanja.bogojevic@imgge.bg.ac.rs](mailto:sanja.bogojevic@imgge.bg.ac.rs) (S.S.B.); [dusan.milivojevic@imgge.bg.ac.rs](mailto:dusan.milivojevic@imgge.bg.ac.rs) (D.M.); [ivana_aleksic@imgge.bg.ac.rs](mailto:ivana_aleksic@imgge.bg.ac.rs) (I.A.)

^3^ University of Ljubljana, Faculty of Chemistry and Chemical Technology, Večna pot 113, SI-1000 Ljubljana, Slovenia; [Franc.Perdih@fkkt.uni-lj.si](mailto:Franc.Perdih@fkkt.uni-lj.si) (F.P.)

^4^ University of Kragujevac, Faculty of Science, Department of Chemistry, R. Domanovića 12, 34000 Kragujevac, Serbia; [tina.andrejevic@pmf.kg.ac.rs](mailto:tina.andrejevic@pmf.kg.ac.rs) (T.A.)

^5^ Serbian Academy of Sciences and Arts, Knez Mihailova 35, 11000 Belgrade, Serbia

*Correspondence: [jasmina.nikodinovic@imgge.bg.ac.rs](mailto:jasmina.nikodinovic@imgge.bg.ac.rs); Tel.: +381 11 397 6034 (J.N.-R.); [biljana.glisic@pmf.kg.ac.rs](mailto:biljana.glisic@pmf.kg.ac.rs); Tel.: +381 34 336 223 (B.Đ.G.); [Iztok.Turel@fkkt.uni-lj.si](mailto:Iztok.Turel@fkkt.uni-lj.si); Tel.: +386 1 47 98 525 (I.T.); [milos.djuran@pmf.kg.ac.rs](mailto:milos.djuran@pmf.kg.ac.rs); Tel.: +381 34 300 251 (M.I.Dj.)

**Abstract:** Three new silver(I) complexes [Ag(NO_3_)(tia)(H_2_O)]*_n_* (**Ag1**), [Ag(CF_3_SO_3_)(1,8-naph)]*_n_* (**Ag2**) and [Ag_2_(1,8-naph)_2_(H_2_O)_1.2_](PF_6_)_2_ (**Ag3**), where tia is thianthrene and 1,8-naph is 1,8-naphthyridine, were synthesized and structurally characterized by different spectroscopic and electrochemical methods and their crystal structures were determined by single-crystal X-ray diffraction analysis. Their antimicrobial potential was evaluated against four bacterial and three *Candida* species, and the obtained results revealed that these complexes showed significant activity toward the Gram-positive *Staphylococcus aureus,* Gram-negative *Pseudomonas aeruginosa* and the investigated *Candida* species with minimal inhibitory concentration (MIC) values in the range 1.56 – 7.81 μg/mL. On the other hand, tia and 1,8-naph ligands were not active against the investigated strains, suggesting that their complexation with Ag(I) ion results in formation of antimicrobial compounds. Moreover, low toxicity of the complexes was detected by *in vivo* model *Caenorhabditis elegans*. The interaction of the complexes with calf thymus DNA (ct-DNA) and bovine serum albumin (BSA) was studied to evaluate their binding affinity towards these biomolecules for possible insights into the mode of antimicrobial activity. The binding affinity of **Ag1 – 3** to BSA was higher than that for DNA, indicating that proteins could be more favorable binding sites for these complexes in comparison to the nucleic acids.

**Keywords:** silver(I) complexes; thianthrene; 1,8-naphthyridine; antimicrobial activity; *Caenorhabditis elegans*; DNA/BSA interaction

**TABLE OF CONTENTS**

| ^1^H NMR spectrum of **Ag1** | 4 |
| --- | --- |
| ^13^C NMR spectrum of **Ag1** | 5 |
| ^1^H NMR spectrum of **Ag2** | 6 |
| ^13^C NMR spectrum of **Ag2** | 7 |
| ^1^H NMR spectrum of **Ag3** | 8 |
| ^13^C NMR spectrum of **Ag3** | 9 |
| ^1^H NMR spectrum of thianthrene | 10 |
| ^13^C NMR spectrum of thianthrene | 11 |
| ^1^H NMR spectrum of 1,8-naphthyridine | 12 |
| ^13^C NMR spectrum of 1,8-naphthyridine | 13 |
| **Figure S1.** Time stability of **Ag3** complex followed by UV-Vis spectrophotometry at room temperature in DMSO. | 14 |
| **Figure S2.** Air/light stability of silver(I) complexes **Ag1 – 3** and corresponding silver(I) salts used for their synthesis. | 15 |
| **Figure S3.** Quantification of interaction of silver(I) complexes **Ag1 – 3** with commercial lambda bacteriophage DNA by gel electrophoresis done in the Excel program. ImageJ program was used for figure analysis. | 16 |
| 1. **Table S1.** Selected bond distances (Å) and valence angles (^o^) in complexes **1** and **2**. | 17 |
| **Experimental data for thianthrene (tia) and 1,8-naphthyridine (1,8-naph)** | 18 |

^1^H NMR spectrum of **Ag1**


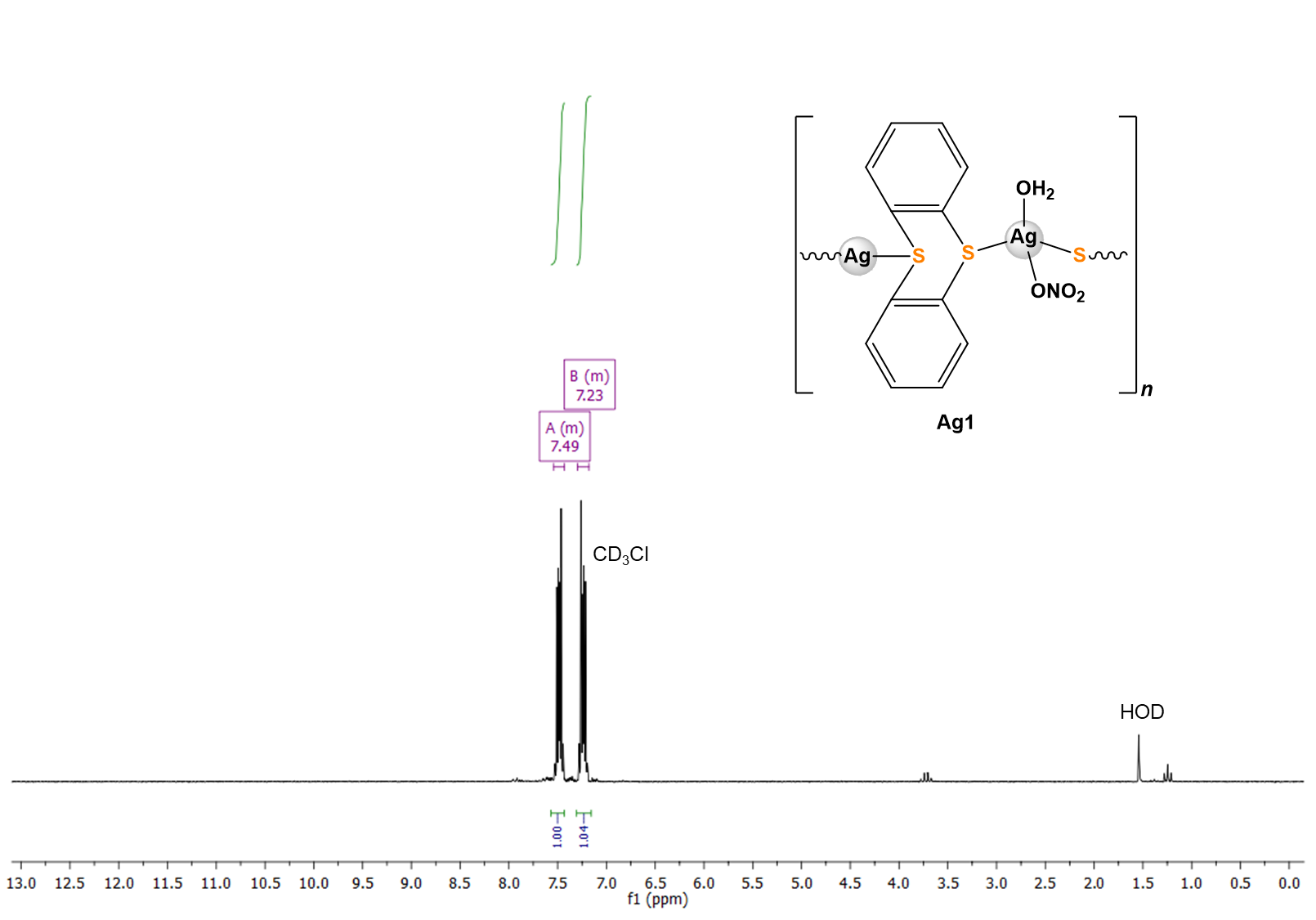


^13^C NMR spectrum of **Ag1**


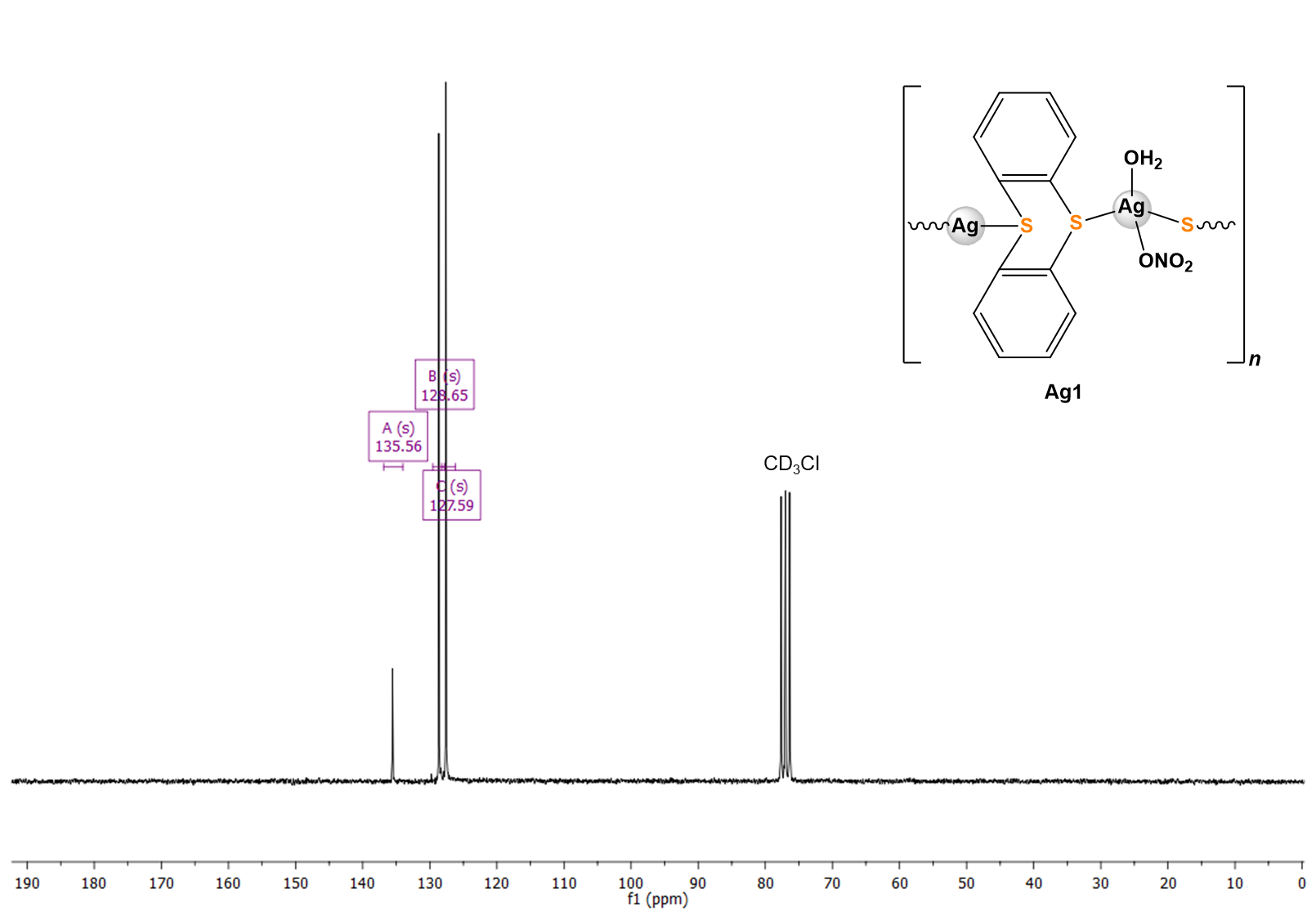


^1^H NMR spectrum of **Ag2**


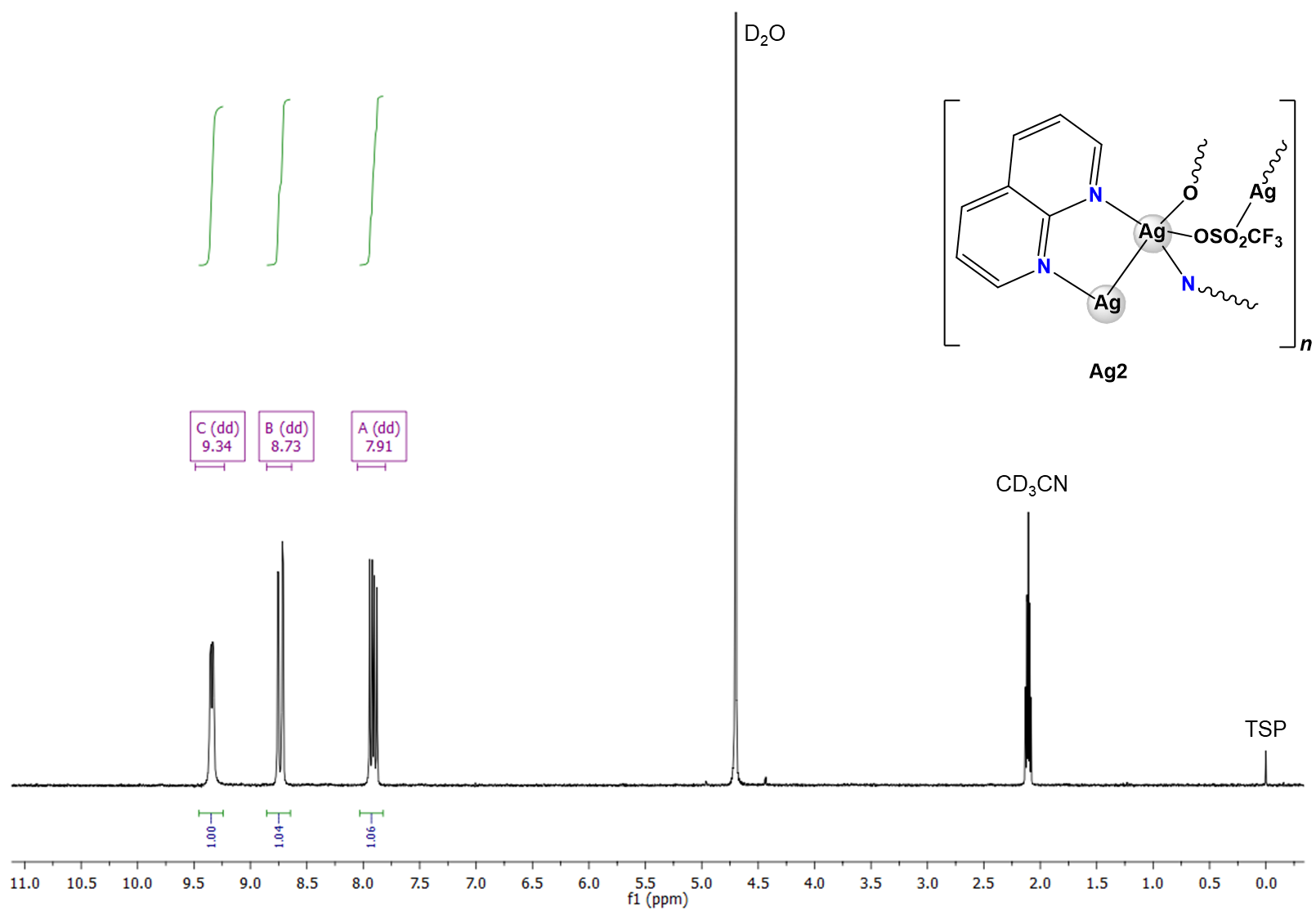


^13^C NMR spectrum of **Ag2**


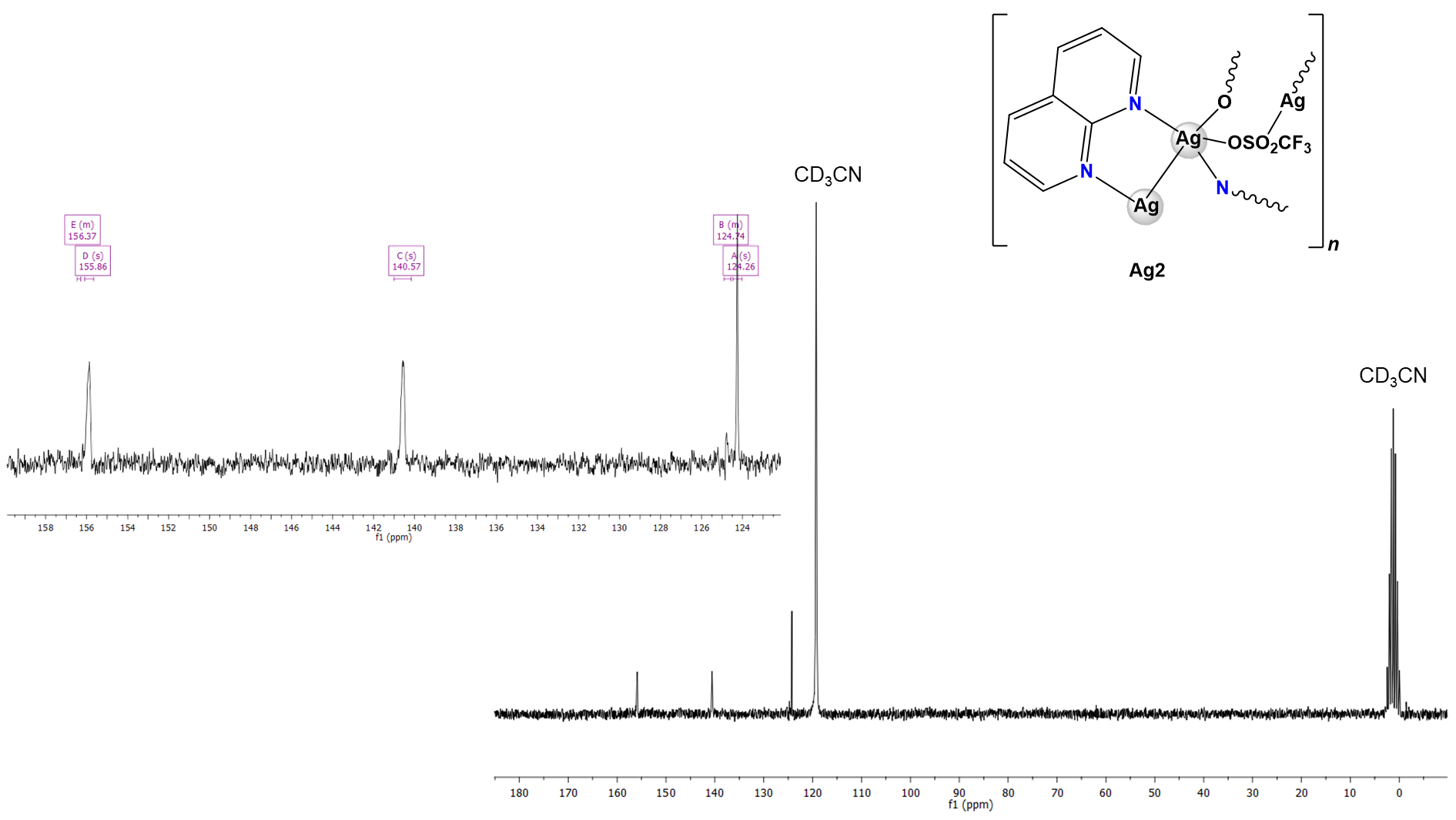


^1^H NMR spectrum of **Ag3**


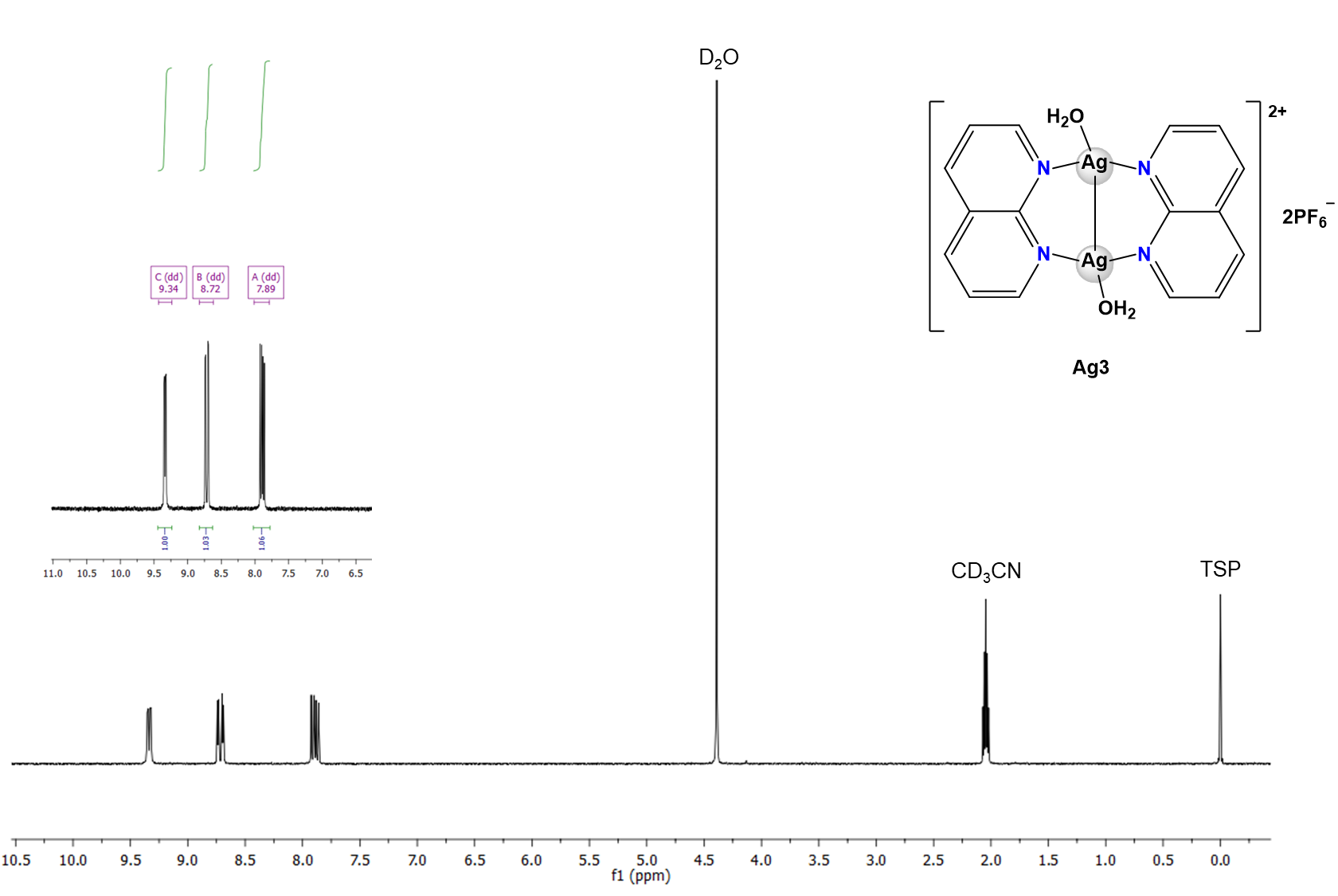


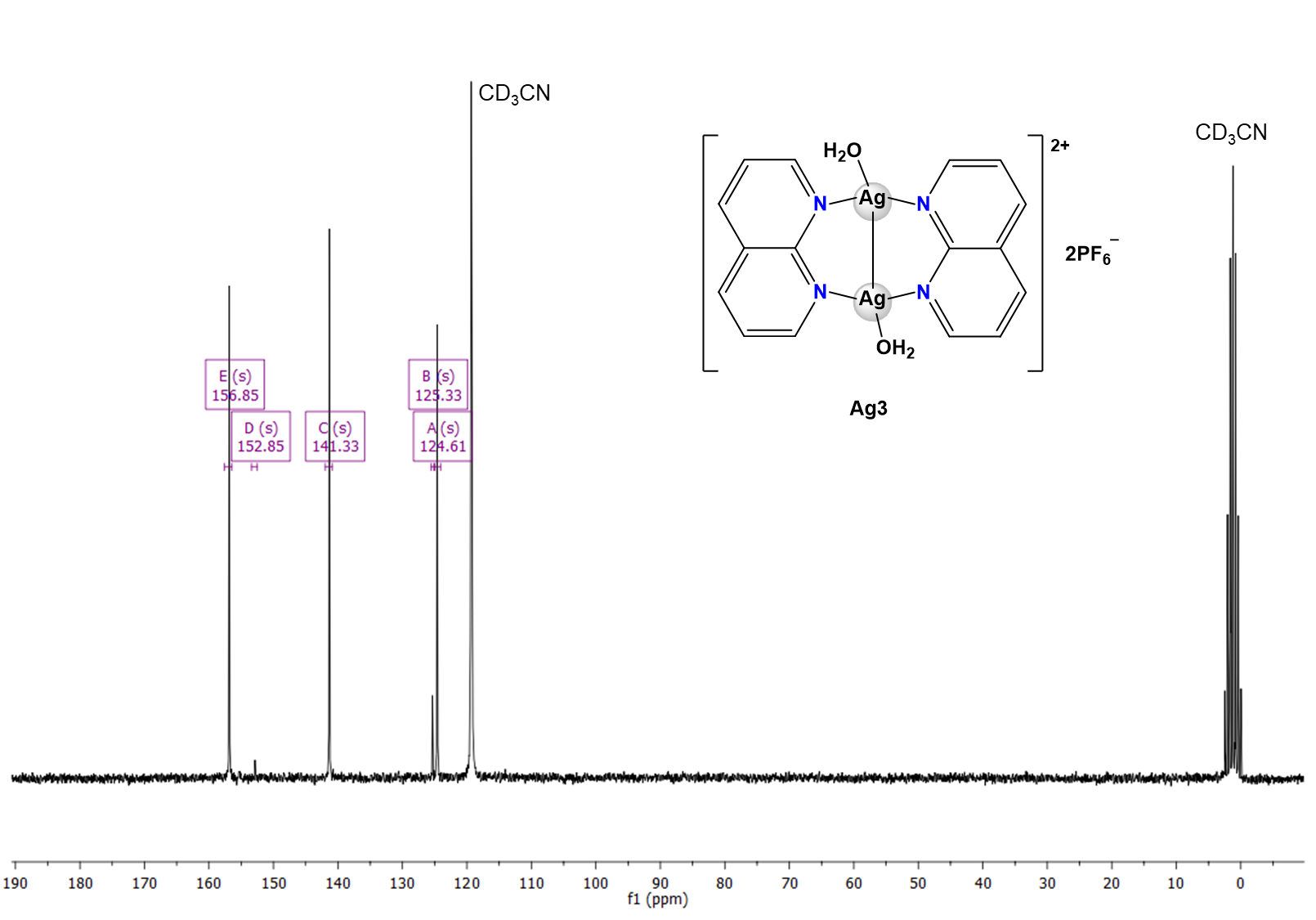
^13^C NMR spectrum of **Ag3**


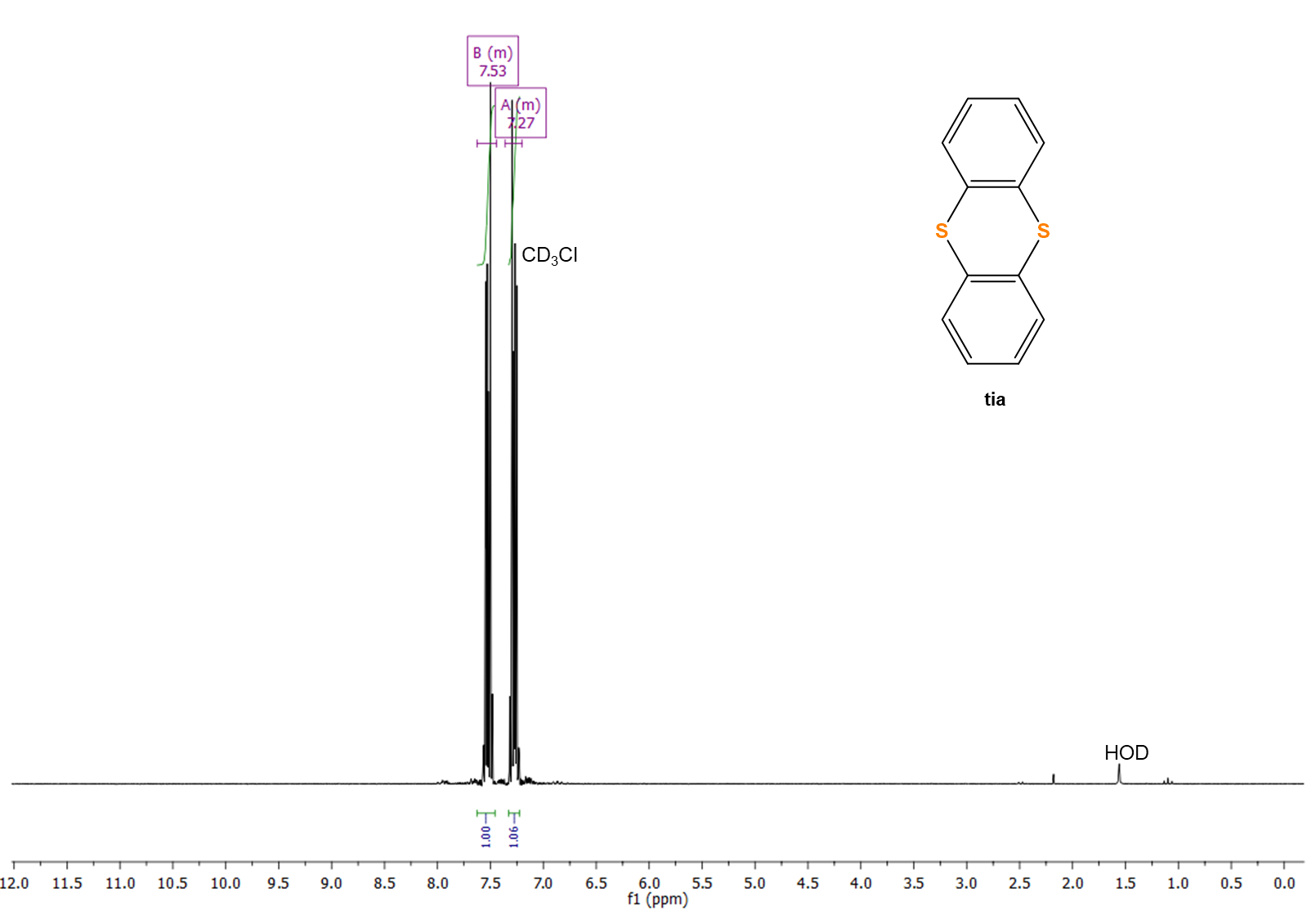
^1^H NMR spectrum of thianthrene


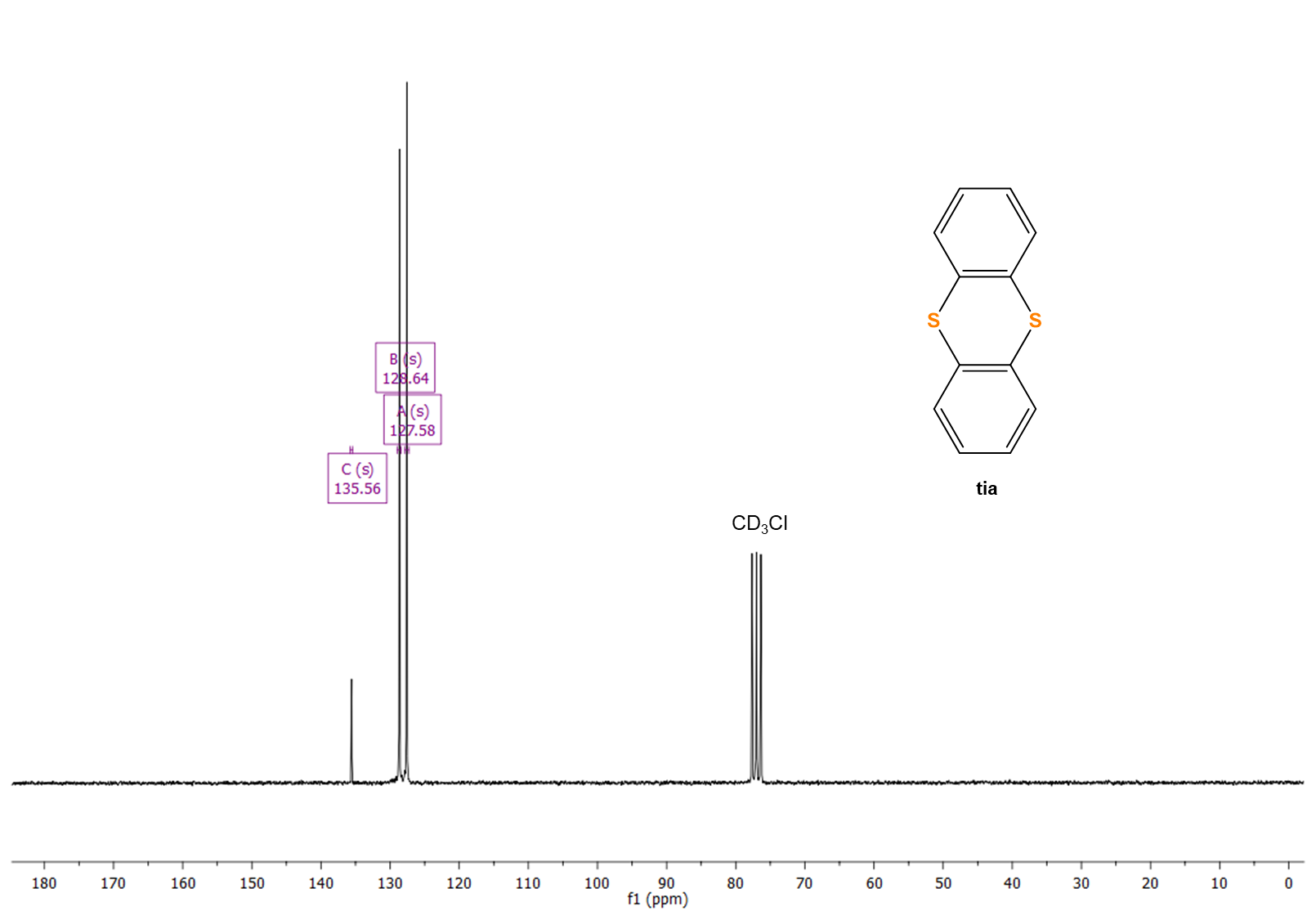
^13^C NMR spectrum of thianthrene

^1^H NMR spectrum of 1,8-naphthyridine


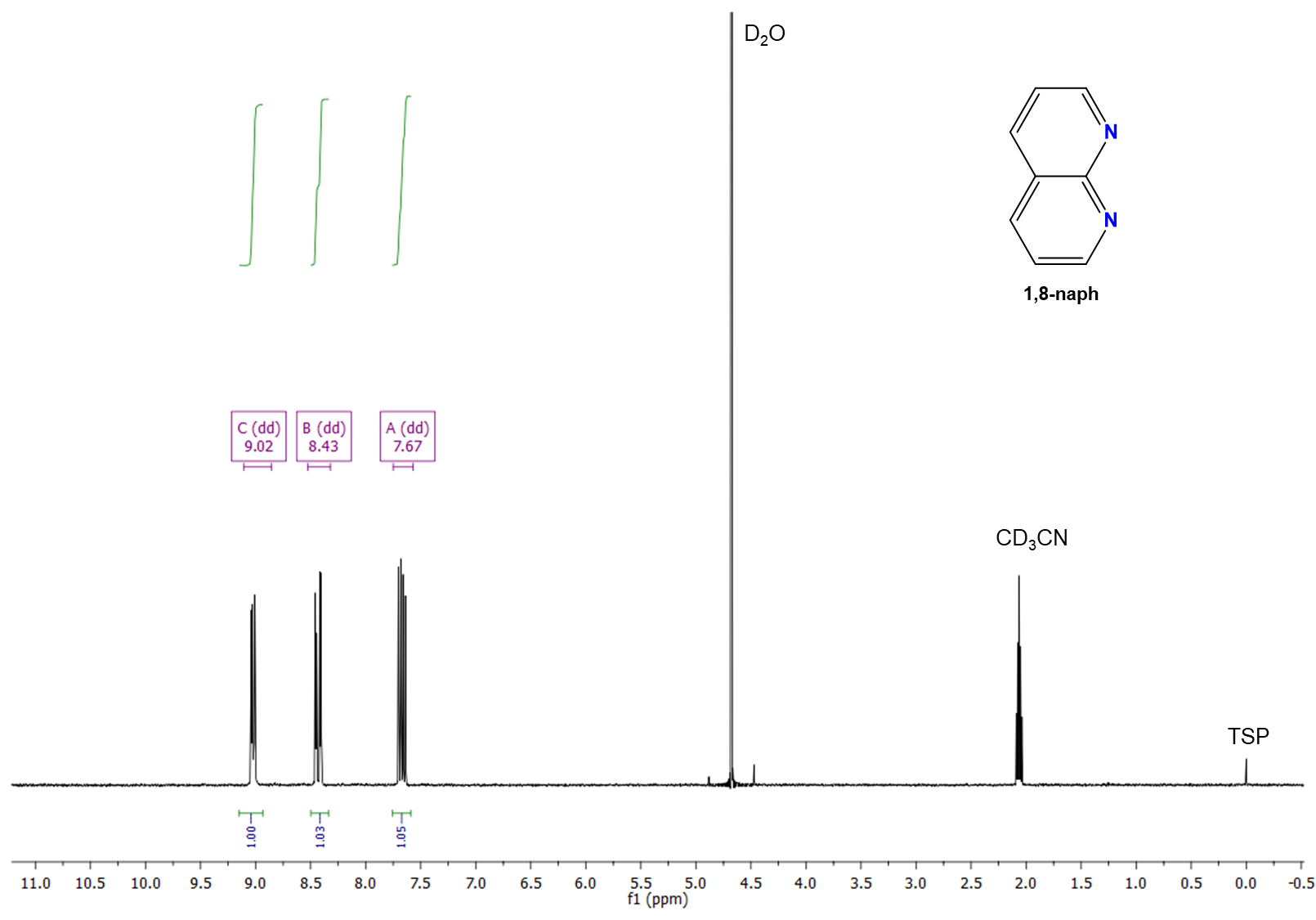

^13^C NMR spectrum of 1,8-naphthyridine


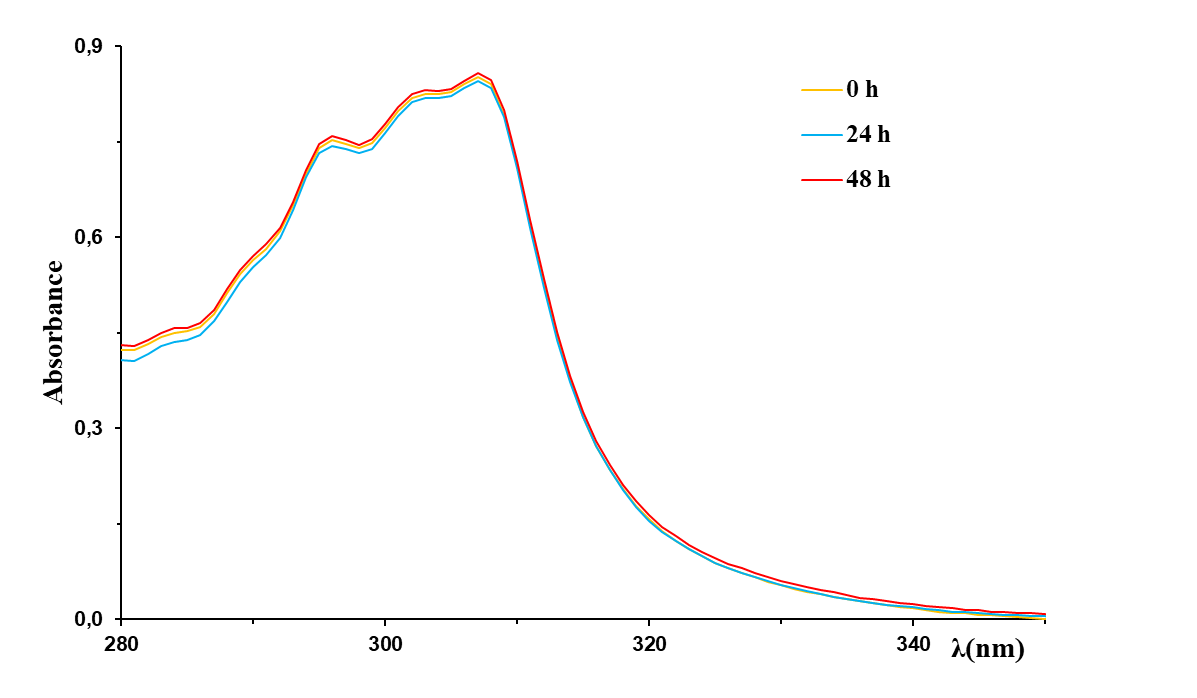


**Figure S1.** Time stability of **Ag3** complex followed by UV-Vis spectrophotometry at room temperature in DMSO.


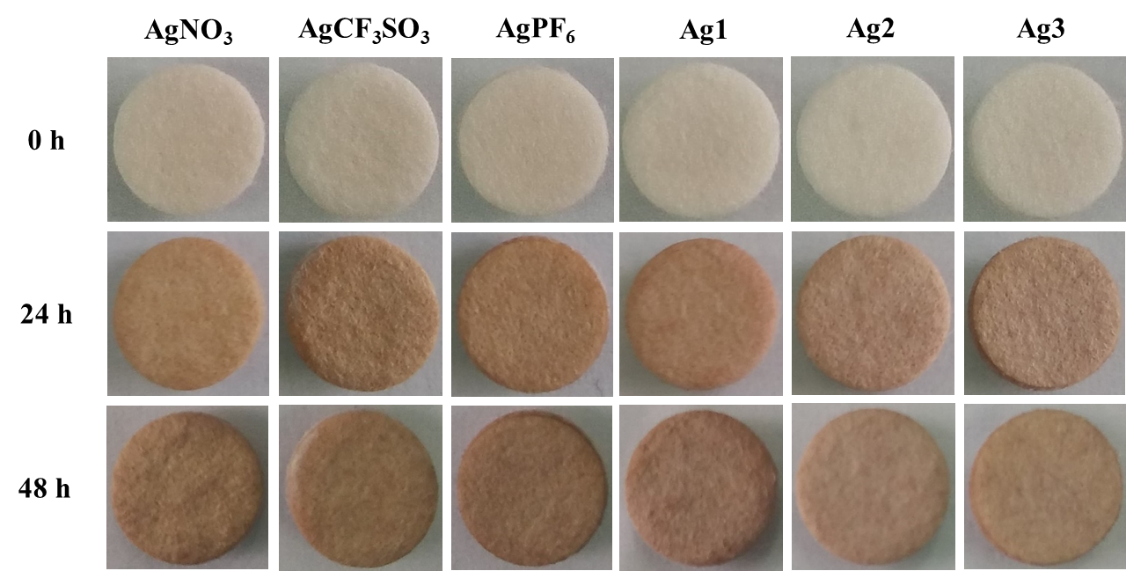


**Figure S2.** Air/light stability of silver(I) complexes **Ag1 – 3** and corresponding silver(I) salts used for their synthesis.


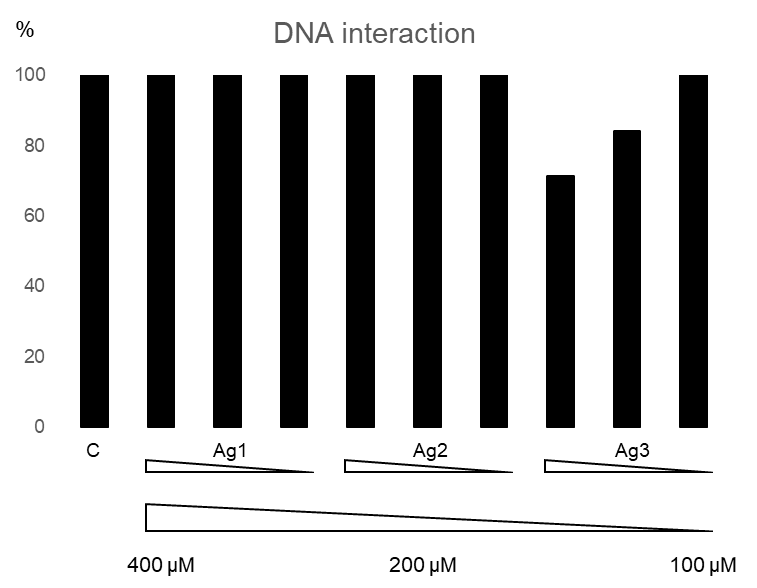


**Figure S3.** Quantification of interaction of silver(I) complexes **Ag1 – 3** with commercial lambda bacteriophage DNA by gel electrophoresis done in the Excel program. ImageJ program was used for figure analysis.

**Table S1.** Details of the crystal structure determination for complexes **Ag1 – 3**.

|  | **Ag1** | **Ag2** | **Ag3** |
| --- | --- | --- | --- |
| CCDC number | 2065519 | 2065520 | 2065521 |
| Formula | C_12_H_10_AgNO_4_S_2_ | C_9_H_6_AgF_3_N_2_O_3_S | C_16_H_14.4_Ag_2_F_12_N_4_O_1.2_P_2_ |
| *M*_r_ | 404.20 | 387.09 | 787.59 |
| *T* (K) | 150.00(10) | 150.00(10) | 150.00(10) |
| Crystal system | monoclinic | monoclinic | triclinic |
| Space group | *P*2_1_/*c* | *P*2_1_/*c* | *P*ī |
| *a* (Å) | 5.7585(4) | 8.5216(4) | 7.5705(5) |
| *b* (Å) | 16.7464(9) | 9.1114(5) | 8.9121(5) |
| *c* (Å) | 14.1677(7) | 15.2361(8) | 9.7311(8) |
| *α* (°) | 90 | 90 | 67.362(7) |
| *β* (°) | 100.300(5) | 102.225(5) | 75.311(6) |
| *γ* (°) | 90 | 90 | 75.443(5) |
| Volume (Å^3^) | 1344.23(14) | 1156.16(11) | 577.40(8) |
| Z | 4 | 4 | 1 |
| *D*_c_ (g/cm^3^) | 1.997 | 2.224 | 2.265 |
| *μ* (mm^–1^) | 1.820 | 1.969 | 1.952 |
| *F*(000) | 800.0 | 752.0 | 380.0 |
| Reflections collected | 6699 | 6303 | 4464 |
| *R*_int_ | 0.0251 | 0.0297 | 0.0408 |
| Data/restraints/parameters | 3094/1/187 | 2642/0/172 | 2648/0/173 |
| *R*, *wR*_2_ [*I* > 2σ(*I*)] *^a^* | 0.0293, 0.0600 | 0.0294, 0.0703 | 1.036 |
| *R*, *wR*_2_ (all data) *^a^* | 0.0372, 0.0653 | 0.0360, 0.0762 | 0.0362, 0.0761 |
| GOF, *S* ^b^ | 1.070 | 1.101 | 0.0439, 0.0832 |
| Largest diff. peak/hole / e/Å^3^ | 0.39/–0.66 | 0.51/–0.93 | 0.90/–0.70 |

*^a^* *R* = ∑||*F*_o_| – |*F*_c_||/∑|*F*_o_|, *wR*_2_ = {∑[*w*(*F*_o_^2^ – *F*_c_^2^)^2^]/∑[*w*(*F*_o_^2^)^2^]}^1/2^. *^b^* *S* = {∑[(*F*_o_^2^ – *F*_c_^2^)^2^]/(*n* – *p*}^1/2^, where *n* is the number of reflections and *p* is the total number of refined parameters.

**Experimental data for thianthrene (tia) and 1,8-naphthyridine (1,8-naph)**

Ligand (tia) data given for comparative purposes. MW = 216.32. ^1^H NMR (200 MHz, CDCl_3_): *δ* = 7.27 (*m*, H2, H3, H7 and H8), 7.53 (*m*, H1, H4, H6 and H9) ppm. ^13^C NMR (50 MHz, CDCl_3_): *δ* = 127.6 (C2, C3, C7 and C8), 128.6 (C1, C4, C6 and C9), 135.6 (C4a, C5a, C9a and C10a) ppm. IR (KBr, ν, cm^-1^): 3063w, 3046w, 3013w (*ν*(C_ar_–H)), 1630w, 1615w, 1552w, 1431m (ν(C_ar_=C_ar_)), 761s (γ(C_ar_–H)), 751s (γ(C–S)). UV-Vis (DMSO, *λ*_max_, nm): 259 (ε = 2.2^.^10^5^ M^-1^cm^-1^).

Ligand (1,8-naph) data given for comparative purposes. MW = 130.15. ^1^H NMR (200 MHz, D_2_O/CD_3_CN): *δ* 7.67 (*dd*, *J* = 8.2, 4.4 Hz, H4 and H5), 8.43 (*dd*, *J* = 8.3, 1.9 Hz, H3 and H6), 9.02 (*dd*, *J* = 4.4, 1.9 Hz, H2 and H7) ppm. ^13^C NMR (50 MHz, D_2_O/CD_3_CN, *v/v* 1:9): *δ* = 123.6 (C4 and C5), 123.8 (C4a), 139.6 (C3 and C6), 154.3 (C2 and C7), 154.7 (C8a) ppm. IR (KBr, ν, cm^-1^): 3098w, 3044w, 3013w (*ν*(C_ar_–H)), 1600m, 1558m, 1490m, 1476w, 1460w, 1394w (ν(C_ar_=C_ar_) and ν(C_ar_=N)), 835s, 809s, 759m (γ(C_ar_–H)). UV-Vis (DMSO, *λ*_max_, nm): 307 (ε = 9.4^.^10^3^ M^-1^cm^-1^).
